# Mannose antagonizes GSDME-mediated pyroptosis through AMPK activated by metabolite GlcNAc-6P

**DOI:** 10.1038/s41422-023-00848-6

**Published:** 2023-07-17

**Authors:** Yuan-li Ai, Wei-jia Wang, Fan-jian Liu, Wei Fang, Hang-zi Chen, Liu-zheng Wu, Xuehui Hong, Yuekun Zhu, Ci-xiong Zhang, Long-yu Liu, Wen-bin Hong, Bo Zhou, Qi-tao Chen, Qiao Wu

**Affiliations:** 1 State Key Laboratory of Cellular Stress Biology, School of Life Sciences, Xiamen University, Xiamen, Fujian China; 2 Department of Gastrointestinal Surgery, Zhongshan Hospital of Xiamen University, School of Medicine, Xiamen University, Xiamen, Fujian China; 3 Department of Colorectal Surgery, The First Affiliated Hospital of Harbin Medical University, Harbin, Heilongjiang China

**Keywords:** Cell death, Chemotherapy

## Abstract

Pyroptosis is a type of regulated cell death executed by gasdermin family members. However, how gasdermin-mediated pyroptosis is negatively regulated remains unclear. Here, we demonstrate that mannose, a hexose, inhibits GSDME-mediated pyroptosis by activating AMP-activated protein kinase (AMPK). Mechanistically, mannose metabolism in the hexosamine biosynthetic pathway increases levels of the metabolite *N*-acetylglucosamine-6-phosphate (GlcNAc-6P), which binds AMPK to facilitate AMPK phosphorylation by LKB1. Activated AMPK then phosphorylates GSDME at Thr6, which leads to blockade of caspase-3-induced GSDME cleavage, thereby repressing pyroptosis. The regulatory role of AMPK-mediated GSDME phosphorylation was further confirmed in AMPK knockout and GSDME^T6E^ or GSDME^T6A^ knock-in mice. In mouse primary cancer models, mannose administration suppressed pyroptosis in small intestine and kidney to alleviate cisplatin- or oxaliplatin-induced tissue toxicity without impairing antitumor effects. The protective effect of mannose was also verified in a small group of patients with gastrointestinal cancer who received normal chemotherapy. Our study reveals a novel mechanism whereby mannose antagonizes GSDME-mediated pyroptosis through GlcNAc-6P-mediated activation of AMPK, and suggests the utility of mannose supplementation in alleviating chemotherapy-induced side effects in clinic applications.

## Introduction

The ubiquitous AMP-activated protein kinase (AMPK), a heterotrimeric protein complex consisting of catalytic subunit α and regulatory subunits β and γ, is a key sensor in cellular energy metabolism.^1,2^ The canonical adenine nucleotide-dependent AMPK activation is induced by cellular energy shortage due to the intracellular depletion of ATP and increase of AMP/ADP levels.^2^ AMP/ADP binds the AMPK γ subunits, triggering a conformational change to enable the phosphorylation of the α subunit at Thr172 by either LKB1 or CaMKK2, two upstream kinases. AMPK can also be activated through compound-dependent noncanonical pathways.^3^ For example, compound A-769662 enables the allosteric activation of AMPK and inhibits the dephosphorylation of AMPK at Thr172 with its binding in between catalytic subunit α and regulatory subunit β.^4^ It was recently demonstrated that a decrease in cellular fructose-1,6-bisphosphate (FBP) also triggered the activation of AMPK through the LKB1-AMPK-Axin signaling pathway on the surface of the lysosome,^5^ suggesting that it was a metabolite that functioned as a signaling molecule to regulate the AMPK activity.

As a metabolic hub, AMPK promotes ATP-generating catabolic processes and inhibits ATP-consuming anabolic processes by phosphorylating a myriad of downstream targets.^1^ Moreover, AMPK has also been linked to many non-metabolic processes, such as those that control the cell fate, including autophagy,^6–10^ apoptosis^10,11^ and ferroptosis.^12^ AMPK abolishes ferroptosis due to ischemia-reperfusion injury by enhancing acetyl-CoA carboxylase (ACC) phosphorylation and inhibiting polyunsaturated fatty acid (PUFA) generation.^12^ A recent study showed that AMPK protects against liver cell death by phosphorylating proapoptotic caspase-6 in nonalcoholic steatohepatitis (NASH),^11^ suggesting the inhibitory effect of AMPK on apoptosis. However, compared to the canonical function of AMPK for metabolic homeostasis, novel substrates of AMPK that determine cell fate and the underlying mechanism remain under investigated.

Pyroptosis is a newly identified form of regulatory cell death. It is characterized by the cleavage of N-terminal segments of gasdermin family members, including GSDMA, GSDMB, GSDMC, GSDMD, and GSDME. Those cleaved N-terminal fragments translocate to and perforate the cell membrane, resulting in cell swelling and cell death.^13^ Our previous study demonstrated that although treatment with some clinical ROS-inducing drugs alone, such as sulfasalazine, could not induce pyroptosis in melanoma cells, co-treatment with iron and sulfasalazine significantly increased the occurrence of GSDME-mediated pyroptosis, in which iron acts as a sensitizer for pyroptotic induction of melanoma cells.^14^ Since melanoma cells are often resistant to apoptosis, this iron-dependent mechanism of pyroptosis induction may represent an alternative therapeutic strategy against melanoma. Despite the benefits of pyroptotic induction in tumor therapy, GSDME is usually silent in most tumor cells but highly expressed in normal cells,^15^ and inappropriate activation of pyroptosis in normal tissues causes tissue damage. Recently, Shao’s and others’ groups revealed that GSDME-mediated pyroptosis is associated with chemotherapy-induced side effects, such as nephrotoxicity,^16,17^ lung injury, and gastrointestinal damage.^15^ Therefore, treatments that can control pyroptosis-induced tissue damage are urgently needed for patients receiving chemotherapeutic agents.

Here, we demonstrate a suppressive effect of mannose on GSDME-mediated pyroptosis by the activation of AMPK. Mannose, a C-2 epimer of glucose,^18^ is not only an effective nonantibiotic drug for urinary tract infections,^19^ but also a potential agent for impairing tumor growth upon combination with conventional chemotherapy.^20^ We found that the metabolism of mannose in the cells elevated the level of the metabolite *N*-acetylglucosamine-6-phosphate (GlcNAc-6P), which bound and activated AMPK. Activated AMPK then phosphorylated GSDME to block caspase-3 binding and cleavage. This finding led to the use of mannose to protect normal organs against chemotherapy-induced injury. In mouse models, the administration of mannose alleviated chemotherapy-induced injury in the small intestine and kidney but did not dampen the antitumor potency of platinum-based chemotherapeutic drugs (cisplatin and oxaliplatin). In eight patients who received chemotherapy, co-adminitration of mannose resulted in the decrease of diarrhea and fecal white blood cells (WBCs). Together, the data presented here demonstrate that GSDME is a novel substrate of AMPK and the activation of AMPK is critical to negatively regulate pyroptosis. It also indicates that mannose supplementation is a simple, safe, and selective option for a combined treatment to relieve the toxic side effects induced by chemotherapy in cancer patients.

## Results

### Mannose reverses GSDME-mediated pyroptosis in different cancer cell lines

**Fig. 1:**
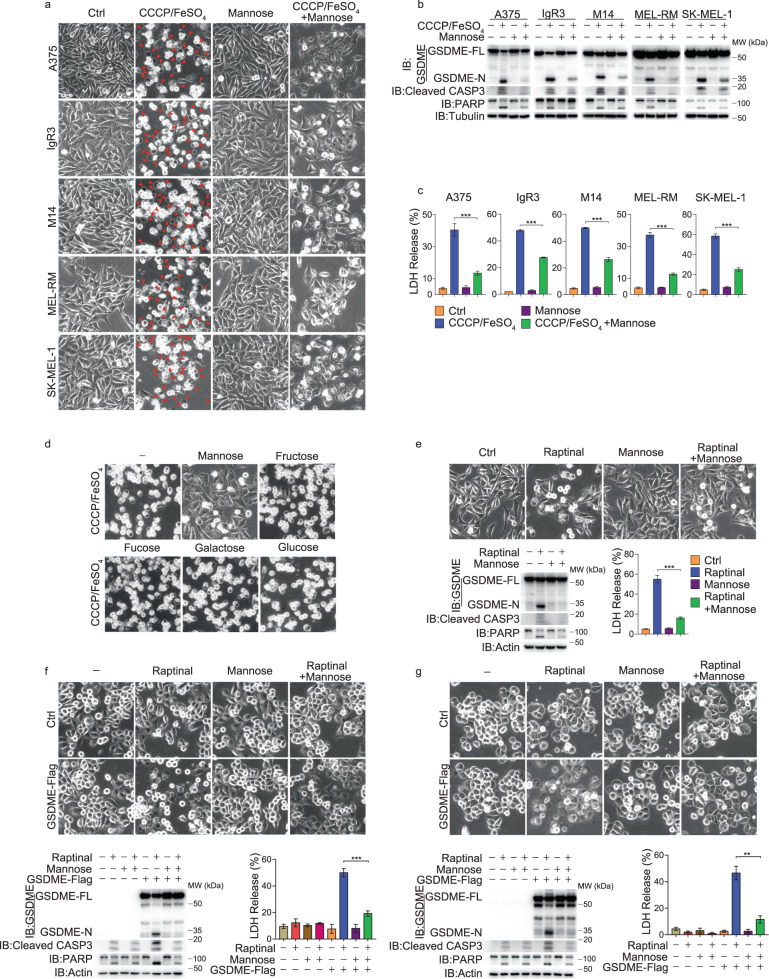
Mannose reverses GSDME-mediated pyroptosis in response to different inducers. Different cancer cell lines were pretreated with mannose (20 mM) for 2 h, followed by different inducers for 24 h to assess pyroptotic features (including characteristic morphology, GSDME cleavage, and LDH release), and the cleaved caspase-3 and its substrate PARP levels were also detected, unless specifically defined. **a**–**c** Different melanoma cell lines were pretreated with mannose, and then with CCCP/FeSO_4_ (CCCP, 20 μM; FeSO_4_, 100 μM). Pyroptosis was detected with morphology (**a**, red arrows indicate pyroptosis cells), GSDME cleavage (**b**), and LDH release (**c**). **d** Mannose, but not other hexoses, reversed CCCP/FeSO_4_-induced pyroptotic morphology. Melanoma A375 cells were pretreated with different reagents (20 mM), including glucose, fructose, fucose and galactose for 2 h. **e** A375 cells were pretreated with mannose, and then with raptinal (1 μM). **f**, **g** GSDME-null cancer cell lines PC-9 cells (**f**) and MDA-MB-468 cells (**g**) were transfected with GSDME first. The cells were then pretreated with mannose, followed by raptinal. Tubulin or actin was used to determine the amount of loading proteins. All data are presented as mean ± SD of two independent experiments, and one of western blotting results is presented. ***P < 0.001, **P < 0.01.

In a previous study, we reported that carbonyl cyanide m-chlorophenyl hydrazine (CCCP) plus iron induced GSDME-dependent pyroptosis in melanoma cells.^14^ In a subsequent study, we found that mannose, a C-2 epimer of glucose, dramatically reversed CCCP/iron-induced pyroptosis (including pyroptotic morphology, GSDME cleavage, and lactate dehydrogenase (LDH) release) in different melanoma cell lines (Fig. 1a–c). However, other hexoses, including glucose, fructose, fucose, and galactose, had no such effects (Fig. 1d), suggesting an unreported role of mannose in suppressing CCCP/iron-induced pyroptosis in melanoma cells.

To test whether mannose would have a general effect on GSDME-dependent pyroptosis, we treated A375 cells with raptinal that was reported to induce GSDME-dependent pyroptosis.^15,21^ The results showed that pyroptosis induced by raptinal was also greatly inhibited by mannose (Fig. 1e). In GSDME-null cancer cell lines, such as human lung adenocarcinoma PC-9 cells and human breast cancer MDA-MB-468 cells, raptinal could not induce pyroptosis. Upon GSDME overexpression in both cell lines, raptinal could induce pyroptosis, which was clearly inhibited by mannose (Fig. 1f, g). However, mannose failed to inhibit pyroptosis mediated by other gasdermins, such as LPS/nigericin-induced GSDMD-dependent pyroptosis in PMA-differentiated THP1 macrophages^22^ and DM-αKG-induced GSDMC-dependent pyroptosis in HeLa cells^23^ (Supplementary information, Fig. S1a, b). As expected, with the extension of treatment time, LDH release and cell death induced by different inducers gradually increased, and further addition of mannose consistently exerted inhibitory effects (Supplementary information, Fig. S1c, d). We also noted that under treatment of different cell death inhibitors, cuproptosis and ferroptosis were slightly involved in CCCP/iron- or raptinal-induced cell death (Supplementary information, Fig. S1e), confirming a dominant role of pyroptosis in the current case. Together, these findings suggest that mannose could be a general inhibitor against GSDME-dependent pyroptosis in various cancerous cell lines.

Although mannose effectively inhibits GSDME cleavage, the expression of full-length GSDME (GSDME-FL) cannot reverse to normal level; we thus evaluated whether CCCP/iron plus mannose treatment affects the stability of GSDME-FL. We showed that GSDME cleavage was not detected under Z-VAD treatment, but the intensity of GSDME-FL band was still decreased a little by CCCP/iron or CCCP/iron/mannose (Supplementary information, Fig. S1f), suggesting that CCCP/iron but not mannose induces GSDME degradation. Furthermore, chloroquine (CQ) but not MG132 restored the level of GSDME-FL (Supplementary information, Fig. S1g), suggesting that CCCP/iron may slightly promote GSDME-FL degradation through autophagy/lysosome pathway, which is not regulated by mannose. Clearly, it could be concluded that mannose suppresses CCCP/iron-induced pyroptosis mainly through inhibiting GSDME cleavage rather than impairing GSDME-FL stability.

### The hexosamine biosynthetic pathway contributes to mannose-reversed pyroptosis

**Fig. 2:**
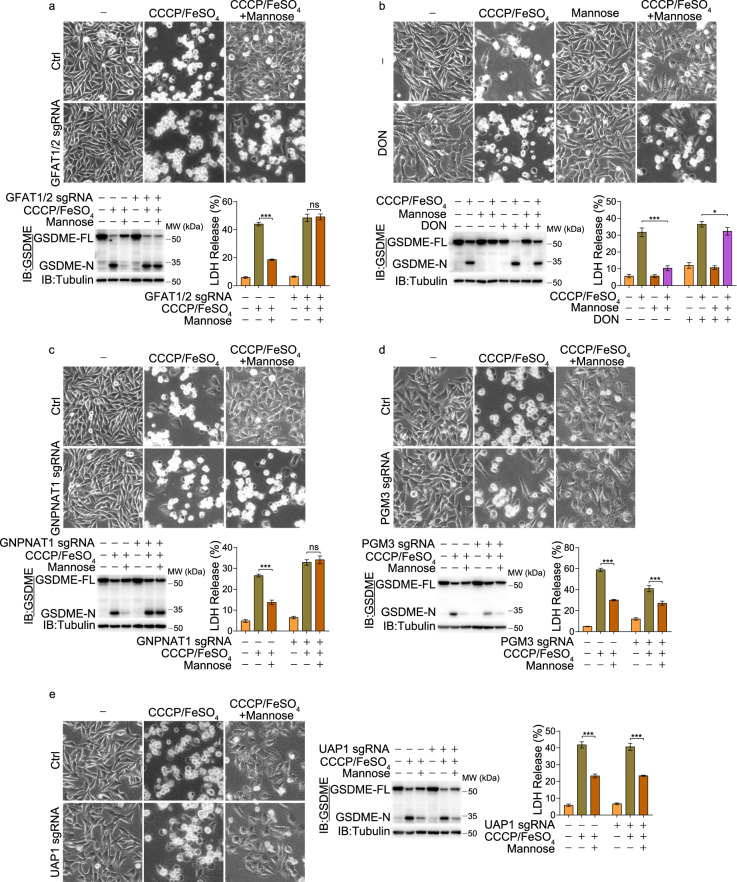
Deletion of genes in HBP impaired the effect of mannose on repressing pyroptosis. Melanoma A375 cells were pretreated with mannose (20 mM) for 2 h, and then CCCP/FeSO_4_ (CCCP, 20 μM; FeSO_4_, 100 μM) for 24 h to assess pyroptotic features (including characteristic morphology, GSDME cleavage, and LDH release), unless specifically defined. **a**, **c**–**e** In GFAT1/2 knockout (**a**), GNPNAT1 knockout (**c**), PGM3 knockout (**d**), and UAP1 knockout (**e**) cells, the effects of mannose on CCCP/FeSO_4_-induced pyroptosis were evaluated. **b** Cells were pretreated with mannose and DON (40 μM) for 2 h as indicated, and pyroptosis was determined. Tubulin was used to determine the amount of loading proteins. All data are presented as mean ± SD of two independent experiments, and one of western blotting results is presented. ***P < 0.001, *P < 0.05; ns not significant.

The metabolism of mannose may be important for the inhibitory effect of mannose on pyroptosis. Mannose and glucose are imported into cells by the same transporters.^18^ Once entering cells, mannose is phosphorylated by hexokinase (HK) to produce mannose-6-phosphate (mannose-6P). Mannose-6P is then either directed to the GDP-mannose biosynthesis pathway or converted to fructose-6-phosphate (F6P), which contributes to the glycolysis pathway and the hexosamine biosynthetic pathway (HBP).^18^ To determine which metabolic pathway is required for mannose to inhibit pyroptosis, the enzymes that catalyze the committed steps in these three pathways were separately knocked out using the CRISPR/Cas9 system (Supplementary information, Fig. S2a). Knocking out phosphomannomutase 2 (PMM2), an enzyme required for the GDP-mannose biosynthesis pathway, affected neither CCCP/iron-induced pyroptosis nor the inhibitory effect of mannose on pyroptosis (Supplementary information, Fig. S2b, c), indicating that the GDP-mannose biosynthesis pathway is not required for mannose to suppress pyroptosis. Phosphofructokinase (PFK) is the rate-limiting enzyme in glycolysis. Among the three isozymes of PFK, PFKM and PFKP were the main isozymes expressed in A375 cells (Supplementary information, Fig. S2b). A double knockout of PFKP and PFKM did not influence the function of mannose in suppressing CCCP/iron-induced pyroptosis (Supplementary information, Fig. S2d), suggesting that the flow of mannose metabolism toward glycolysis is dispensable for the inhibitory effect of mannose on pyroptosis. Similarly, knocking out glucose-6-phosphate isomerase (GPI), another enzyme in the glycolysis pathway, did not influence the inhibitory effect of mannose on pyroptosis (Supplementary information, Fig. S2e). In contrast, a double knockout of glutamine fructose-6-phosphate aminotransferase 1 and 2 (GFAT1/2), the first rate-limiting enzyme in HBP, or a treatment with 6-Diazo-5-oxo-L-norleucine (DON), a GFAT inhibitor, basically abolished the inhibitory effect of mannose on pyroptosis (Fig. 2a, b), which suggests that the flow of mannose metabolism toward HBP may be a prerequisite for the suppressive effect of mannose on pyroptosis.

To further dissect the metabolic steps in HBP involved in pyroptosis suppression, other enzymes in HBP were separately knocked out. Knockout of glucosamine-phosphate *N*-acetyltransferase 1 (GNPNAT1) greatly abolished the suppressive effect of mannose on CCCP/iron-induced pyroptosis (Fig. 2c). However, knockout of either phosphoglucomutase 3 (PGM3) or UDP-*N*-acetylglucosamine pyrophosphorylase 1 (UAP1) did not influence the suppressive effect of mannose on pyroptosis (Fig. 2d, e). Therefore, GFAT and GNPNAT1 in HBP are responsible for the function of mannose in reversing the CCCP/iron-induced pyroptosis.

### Mannose induces AMPK phosphorylation to attenuate pyroptosis

**Fig. 3:**
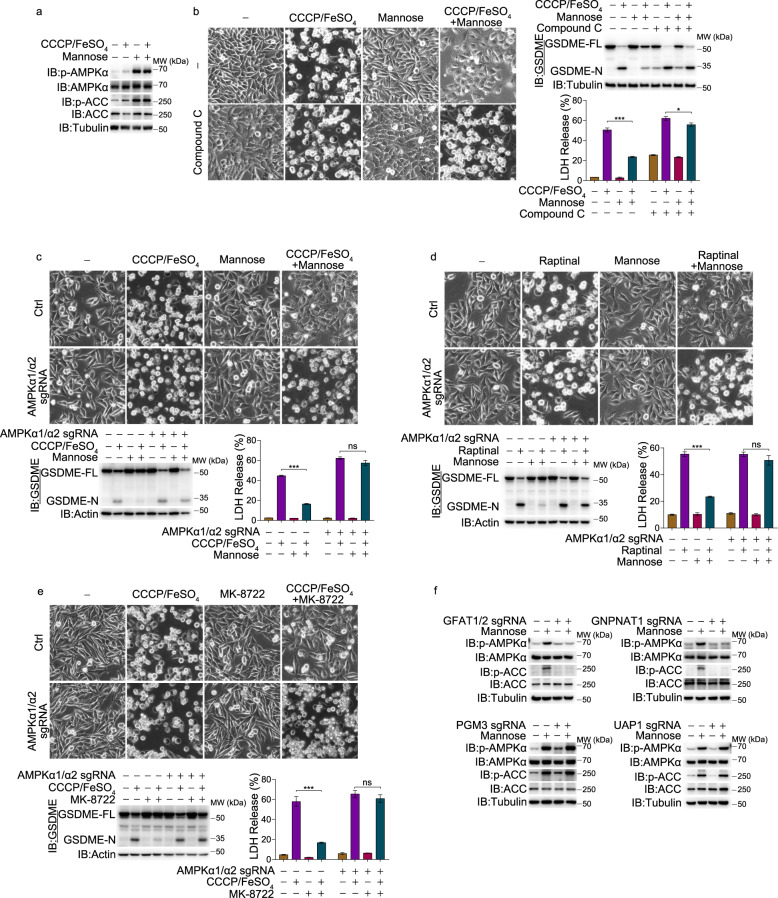
Activation of AMPK mediates repression of pyroptosis by mannose. Melanoma A375 cells were pretreated with mannose (20 mM) for 2 h, and then CCCP/FeSO_4_ (CCCP, 20 μM; FeSO_4_, 100 μM) for 6 h to detect AMPK and ACC phosphorylation, or for 24 h to assess pyroptotic features (including characteristic morphology, GSDME cleavage, and LDH release), unless specifically defined. **a** Cells were treated as described above, and the phosphorylation levels of AMPK (Thr172) and ACC (Ser79) were determined. **b** Cells were pretreated with compound C (12.5 μM) for 2 h, and pyroptosis was then detected. **c**–**e** AMPKα1/α2 knockout cells were pretreated with mannose (**c**, **d**) or MK-8722 (**e**, 1 μM) for 2 h, followed by CCCP/FeSO_4_ (**c**, **e**) or raptinal (**d**), respectively, and pyroptosis was then assayed. **f** Different genes as indicated were knocked out, respectively, and the phosphorylation levels of AMPK and ACC were determined. Tubulin or actin was used to determine the amount of loading proteins. All data are presented as mean ± SD of two independent experiments, and one of western blotting results is presented. ***P < 0.001, *P < 0.05; ns not significant.

Recently, it was reported that mannose treatment was associated with the activation of AMPK in osteosarcoma cells.^20^ Here, we also found that in A375 cells, mannose alone or together with CCCP/iron greatly increased AMPK phosphorylation at Thr172, which was associated with the phosphorylation of the AMPK downstream factor ACC (Fig. 3a). This AMPK activation seemed to be specifically induced by mannose but not any other hexoses (Supplementary information, Fig. S3a), consistent with the results shown in Fig. 1d.

Whether mannose-activated AMPK is associated with pyroptosis inhibition has not been reported. To test this, different concentrations of mannose were used to treat cells. With increasing concentrations of mannose, AMPK phosphorylation was gradually increased (Supplementary information, Fig. S3b), thereby leading to inhibition of CCCP/iron-induced pyroptosis (Supplementary information, Fig. S3c). Furthermore, upon pretreatment of A375 cells with compound C (an AMPK inhibitor^24^), mannose could no longer suppress CCCP/iron-induced pyroptosis (Fig. 3b). Double knockout of AMPKα1 and α2 also abolished the inhibitory effect of mannose on pyroptosis induced by CCCP/iron or raptinal (Fig. 3c, d; Supplementary information, Fig. S3d). Notably, AMPK activation was also sufficient to suppress pyroptosis because the activation of AMPK by its activator MK-8722 markedly suppressed CCCP/iron-induced pyroptosis. In contrast, if both AMPKα1 and α2 were knocked out, MK-8722 lost its repressive effect on pyroptosis (Fig. 3e). Therefore, mannose inhibits pyroptosis by activating AMPK.

The AMP/ATP and ADP/ATP ratios are associated with the activation of AMPK.^2^ However, mannose treatment influence neither the AMP/ATP ratio nor the ADP/ATP ratio (Supplementary information, Fig. S3e). Given that HBP is crucial for the suppression of pyroptosis by mannose, it is possible that HBP is also required for the AMPK activation. Pretreatment with DON indeed abolished mannose-induced AMPK activation in different melanoma cell lines (Supplementary information, Fig. S3f). In A375 cells, knockout of either GFAT1/2 or GNPNAT1 also attenuated the phosphorylation of AMPK induced by mannose; in contrast, knockout of either PGM3 or UAP1 did not affect mannose-induced AMPK phosphorylation (Fig. 3f). Collectively, these results not only demonstrate a novel role for AMPK activation in pyroptotic repression but also implicate HBP in mannose-induced AMPK activation.

### The metabolite GlcNAc-6P in HBP targets and activates AMPK

**Fig. 4:**
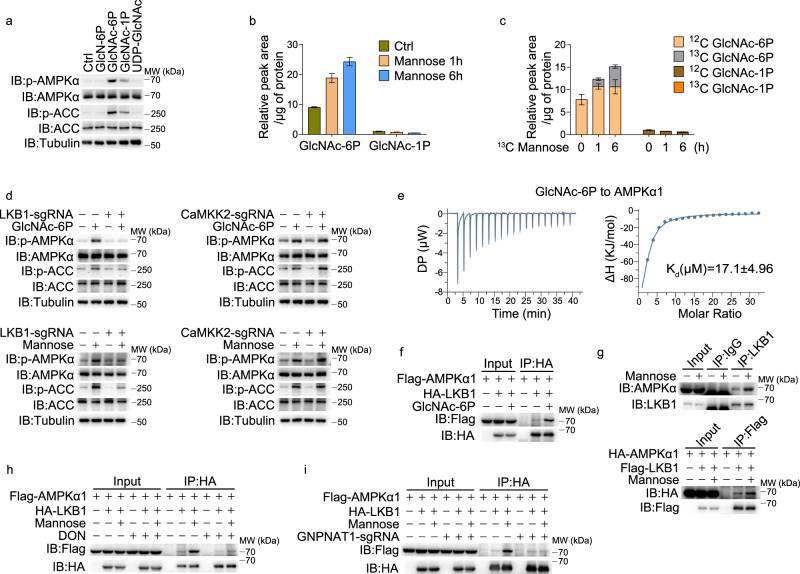
GlcNAc-6P directly binds to and activates AMPKα. **a** SLO (200 ng/mL) and different metabolites (1 mM) as indicated were incubated with A375 cells for 10 min. The phosphorylation levels of AMPK and ACC were detected. **b** Intracellular metabolites were extracted from A375 cells, and the peak areas of GlcNAc-6P and GlcNAc-1P were measured by LC-MS. **c** A375 cells were incubated with ^12^C_6_-glucose medium supplemented with ^13^C_6_-mannose (20 mM). Intracellular metabolites were extracted and measured by LC-MS. **d** LKB1 or CaMKK2 knockout A375 cells were incubated with SLO and GlcNAc-6P (top) or treated with mannose (bottom). The phosphorylation levels of AMPK and ACC were detected. **e** Interaction of purified AMPKα1 and GlcNAc-6P analyzed by ITC. Raw calorimetric data and binding isotherm of the interaction of molecule and protein are shown. **f** A375 cells were incubated with SLO and GlcNAc-6P for 10 min, and co-IP assay was performed. **g** A375 cells were treated with mannose for 6 h, and then co-IP assay of endogenous (top) or exogenously transfected (bottom) proteins was performed. **h**, **i** In DON-treated (**h**) or GNPNAT1 knockout (**i**) A375 cells, co-IP assay was performed. Tubulin was used to determine the amount of loading proteins. All data are presented as mean ± SD of two independent experiments, and one of western blotting results is presented.

The mechanism underlying this mannose-induced AMPK activation was further explored. As HBP was required for mannose-induced AMPK activation (Fig. 3) and mannose did not influence the mRNA or protein levels of enzymes in HBP (Supplementary information, Fig. S4a), we hypothesized that metabolites in HBP might function as signaling molecules to activate AMPK. Since metabolites in HBP hardly go through the cell membrane by incubation, A375 cells were permeabilized with streptolysin O (SLO) before incubation with different intermediates. Incubation with GlcNAc-6P substantially elevated AMPKα phosphorylation, and GlcNAc-1P had a slight effect on AMPK activation, while other metabolites in HBP, such as glucosamine-6P and UDP-GlcNAc failed to activate AMPK (Fig. 4a). In addition, F6P and FBP, intermediates in the glycolysis pathway, had no obvious effect on AMPK activity (Supplementary information, Fig. S4b). Therefore, GlcNAc-6P and GlcNAc-1P are likely required for the mannose-induced AMPK activation.

It was essential to explore whether these HBP intermediates were derived from mannose. We showed that mannose treatment markedly elevated the level of GlcNAc-6P in A375 cells, as detected by liquid chromatography coupled with mass spectrometry (LC-MS) analysis (Fig. 4b; Supplementary information, Fig. S4c). Furthermore, we measured the levels of these two metabolites when cells were incubated with ^12^C_6_-glucose medium supplemented with ^13^C_6_-mannose. The carbon atoms derived from ^13^C_6_-mannose were clearly incorporated into GlcNAc-6P, resulting in an elevated level of GlcNAc-6P, although glucose also partially contributed to GlcNAc-6P accumulation (Fig. 4c). Hence, the metabolism of mannose in HBP leads to the elevation of GlcNAc-6P, which activates AMPK.

The upstream kinases LKB1 and CaMKK2 phosphorylate Thr172 in the α subunit of AMPK to activate AMPK.^2^ Knockout of LKB1 but not CaMKK2 abolished the effect of GlcNAc-6P or mannose on AMPKα phosphorylation (Fig. 4d), suggesting the requirement of LKB1 for mannose-induced AMPK activation through elevation of GlcNAc-6P. Isothermal titration calorimetry (ITC) assays showed that GlcNAc-6P directly bound his-AMPKα1 (*K*_d_ = 17.1 μM, Fig. 4e) but not AMPKβ (Supplementary information, Fig. S4d). Docking analysis further suggests that three amino acid residues of AMPKα1 (K40, K42 and N59, evolutionally conserved sites) may be responsible for GlcNAc-6P binding through the formation of hydrogen bonds (Supplementary information, Fig. S4e). When these residues were mutated individually or combinedly, mannose could no longer activate AMPKα, and GlcNAc-6P failed to bind with these AMPKα1 mutants (Supplementary information, Fig. S4f, g). Based on these findings, we speculated that the binding of GlcNAc-6P to AMPKα1 may influence the interaction of AMPKα1 with LKB1. Indeed, the interaction of AMPKα1 with LKB1 was markedly increased by incubation with GlcNAc-6P (Fig. 4f), and 500 μM GlcNAc-6P was sufficient to enhance this interaction and then activated AMPK phosphorylation (Supplementary information, Fig. S4h, i). Mannose treatment also elevated the AMPKα1–LKB1 interaction (Fig. 4g), and this mannose-enhanced interaction could be abolished by either DON treatment (Fig. 4h) or GNPNAT1 knockout (Fig. 4i), indicating that the mannose-induced enhancement of the AMPKα1–LKB1 interaction is dependent on HBP. Together, this series of results demonstrated that the flow of mannose metabolism toward HBP to elevate GlcNAc-6P is a prerequisite for AMPK activation, and the binding of GlcNAc-6P to AMPKα further facilitates the interaction of AMPKα with the upstream kinase LKB1, thereby leading to the phosphorylation of AMPKα.

Glucosamine (GlcN) and *N*-acetylglucosamine (GlcNAc) can also be metabolized into GlcNAc-6P within cells. However, stimulation of neither GlcN nor GlcNAc induced AMPK phosphorylation, LKB1–AMPK interaction and pyroptosis suppression (Supplementary information, Fig. S4j–l). To investigate the reason, we carried out TurboID proximity labeling assays to identify proteins associated with AMPKα1, and found that GFAT1 and GNPNAT1 (two sequential enzymes of HBP), but not HK (responsible for the phosphorylation of GlcN to produce GlcN-6P)^25^ or GNK (*N*-acetyl-D-glucosamine kinase, which phosphorylates GlcNAc to generate GlcNAc-6P),^26^ were among the list of the AMPKα1 binding partners (Table 1; Supplementary information, Table S1 and Fig. S4m). The complex of AMPKα1, AMPKβ1, AMPKγ1, GFAT1 and GNPNAT1 was further verified by co-immunoprecipitation (co-IP) assays (Supplementary information, Fig. S4n), indicating that this complex might form metabolon that facilitates GlcNAc-6P generation locally. Notably, phosphomannose isomerase (PMI) was also found to be involved in the complex with AMPK (Supplementary information, Fig. S4n). In addition, TurboID and co-IP assays showed that tubulin interacted with AMPKα1 and GNPNAT1, implying that microtubule may participate in the formation of the AMPK complex. When A375 cells were treated with monomethyl auristatin E (MMAE), a depolymerization reagent of microtubule, the associations of AMPKα1 with PMI, GFAT1 and GNPNAT1 were obviously diminished (Supplementary information, Fig. S4o). Under this circumstance, mannose could hardly activate AMPK phosphorylation (Supplementary information, Fig. S4p, top). As a control, the activation of AMPK by MK-8722 was not influenced by MMAE (Supplementary information, Fig. S4p, bottom). It is likely that the association of HBP metabolon with AMPK (the AMPK/PMI/GFAT1/GNPNAT1 complex) might generate high concentration of GlcNAc-6P locally to activate AMPK compartmentally. However, the lack of AMPK/GNPNAT1/HK1 or AMPK/GNK complex may cause insufficient local GlcNAc-6P concentration, leading to inactivation of AMPK by GlcN or GlcNAc.

**Table 1:** Statistics of pathway enrichment.

Pathway	Count	Description
AMPK signaling pathway	6	STK11, AMPKβ2, AMPKβ1, AMPKγ1, AMPKα1, AMPKγ2
p53 pathway	4	P53, P73, SUMO3, HDAC1
FoxO signaling pathway	4	PLK1, HOMER1, STK11, USP7
mTOR signaling pathway	3	CAB39, PIK3R1, RICTOR
UDP-*N*-acetyl-D-glucosamine biosynthesis	2	GFAT1, GNPNAT1

### AMPK phosphorylates GSDME to block caspase-3-mediated cleavage

**Fig. 5:**
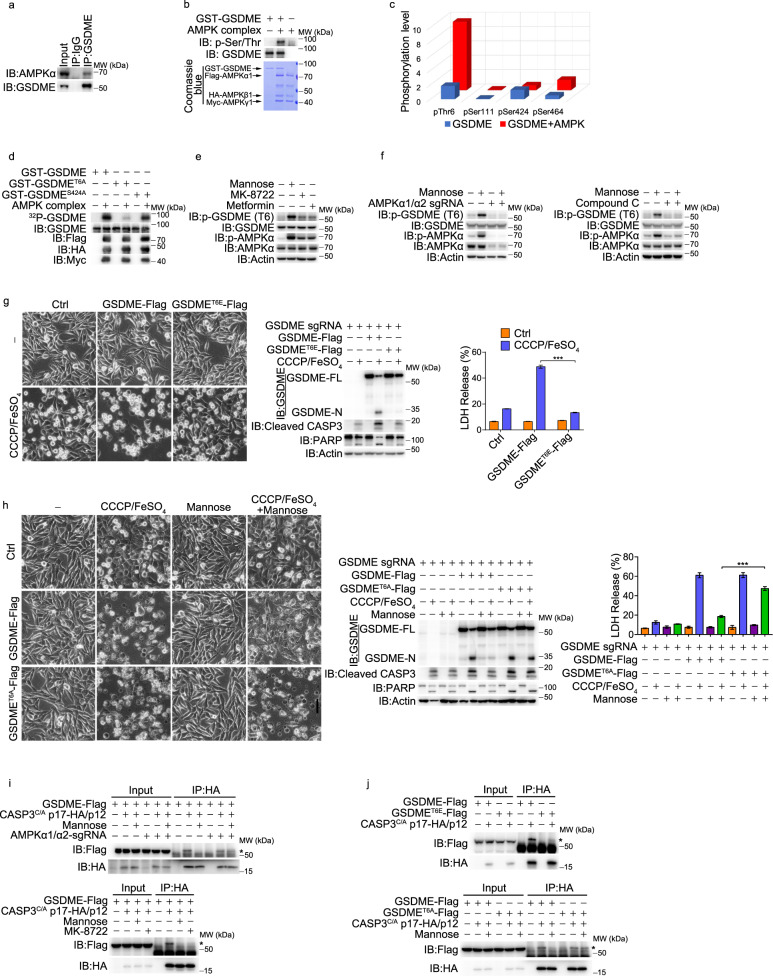
AMPK phosphorylates GSDME to block caspase-3-mediated cleavage. Melanoma A375 cells were pretreated with mannose (20 mM) for 2 h, and then CCCP/FeSO_4_ (CCCP, 20 μM; FeSO_4_, 100 μM) for 24 h to assess pyroptotic features (including characteristic morphology, GSDME cleavage, and LDH release), and the cleaved caspase-3 and its substrate PARP levels were also detected, unless specifically defined. **a** Co-IP assay showed the interaction of endogenous AMPK with GSDME. **b** AMPK directly phosphorylated GSDME in the in vitro AMPK kinase assay. AMPK complex was expressed in HEK293T and purified by HA antibody; GST-GSDME was expressed in *E. coli* strain BL21 (DE3) and purified by GST pull-down. Reactions were resolved by SDS-PAGE and detected by phospho-antibody. **c** LC-MS/MS profiling of the in vitro AMPK kinase assay showed that GSDME Thr6 is specifically phosphorylated by AMPK. **d** Active AMPK was incubated with either GSDME, GSDME T6A or S424A in the in vitro AMPK kinase assay with ^32^P-labeled ATP. Reactions were resolved by SDS-PAGE and detected by autoradiograph. **e** Cells were treated with mannose, MK-8722 (1 μM) or metformin (1 mM) for 6 h. GSDME was immunoprecipitated using anti-GSDME antibody, and then the phosphorylation levels of GSDME T6 were detected. **f** AMPKα1/α2 knockout (left) and compound C-pretreated A375 cells (right) were administered with mannose for 6 h. GSDME was immunoprecipitated using anti-GSDME antibody, and then the phosphorylation levels of GSDME T6 were detected. **g**, **h** In GSDME knockout cells, GSDME and its point mutant T6E or T6A were separately transfected into cells, and then pyroptosis was detected. **i** Mannose or MK-8722 (1 μM) were used to treat WT or AMPKα1/α2 knockout A375 cells for 6 h, and the interactions between GSDME and CASP3^C/A^p17-HA/p12 were determined. The asterisks indicate co-immunoprecipitated GSDME-Flag. **j** Cells were transfected with different plasmids as indicated, and the interactions between GSDME mutants and CASP3^C/A^p17-HA/p12 were determined. Actin was used to determine the amount of loading proteins. All data are presented as mean ± SD of two independent experiments, and one of western blotting results is presented. ***P < 0.001.

The mechanism underlying the effect of AMPK on caspase-3-mediated GSDME cleavage was further investigated. We found that AMPKα did not interact with caspase-3 (Supplementary information, Fig. S5a). However, the interaction of AMPKα with endogenous or transfected GSDME was clearly detected in A375 cells, which was further verified in vitro (Fig. 5a; Supplementary information, Fig. S5b). We therefore speculated that AMPK, a protein kinase, may phosphorylate and regulate GSDME. Indeed, this interaction led to AMPK-mediated phosphorylation of GSDME, as revealed by in vitro phosphorylation assays (Fig. 5b). GSDME contains four putative AMPK phosphorylation sites, Thr6, Ser111, Ser424 and Ser464 (Supplementary information, Fig. S5c). Quantitative mass spectrometric analysis revealed that the incubation with the AMPK complex could induce robust phosphorylation of GSDME at Thr6 but not at Ser111, Ser424, or Ser464 (Fig. 5c; Supplementary information, Fig. S5d). In radioactive in vitro kinase assays, when Thr6 was mutated to Ala, AMPK-induced GSDME phosphorylation was greatly diminished. In contrast, the mutation of Ser424 to Ala did not influence AMPK-induced GSDME phosphorylation (Fig. 5d). We further generated an anti-phospho-Thr6 GSDME antibody (p-GSDME(T6)) and verified that AMPK phosphorylated GSDME but not GSDME^T6A^ (Supplementary information, Fig. S5e). Moreover, the phosphorylation of GSDME at Thr6 was greatly increased by mannose and moderately increased by the AMPK activator MK-8722 and metformin (Fig. 5e). However, when endogenous AMPKα was knocked out or inhibited by compound C, mannose lost its ability to induce the phosphorylation of GSDME at Thr6 (Fig. 5f). Clearly, AMPK induces the phosphorylation of GSDME at Thr6 in response to mannose stimulation. In GSDME-knockout A375 cells, the transfection of GSDME^T6E^ (a phosphorylation mimic mutant), but not GSDME^S111E^, GSDME^S424E^ or GSDME^S464E^, attenuated CCCP/iron-induced pyroptosis (Fig. 5g; Supplementary information, Fig. S5f). In contrast, the transfection of GSDME^T6A^ (a nonphosphorylatable mutant) led to partial loss of mannose ability to suppress CCCP/iron-induced pyroptosis (Fig. 5h). These results suggest that the phosphorylation of GSDME at Thr6 is required for mannose to suppress GSDME-dependent pyroptosis. The facts that GSDME^T6E^ mutant alone or GSDME from mannose-stimulated A375 cells was resistant to recombinant human caspase-3 (rhCASP3)-mediated cleavage in vitro (Supplementary information, Fig. S5g, h) indicate that the cleavage of GSDME by recombinant caspase-3 is almost abolished when GSDME Thr6 was mutated into Glu. Thus, it is possible that the phosphorylation of GSDME at Thr6 by AMPK directly influences the recognition of GSDME by caspase-3. To address this, we constructed a heterodimeric enzyme complex of caspase-3 with a catalytically inactive mutant (referred to as CASP3^C/A^p17-HA/p12). GSDME interacted with CASP3^C/A^p17-HA/p12 but not full-length CASP3^C/A^FL-HA (Supplementary information, Fig. S5i), which was blocked by mannose and MK-8722. However, AMPKα1/α2 knockout would cause mannose to lose this effect (Fig. 5i). Consistently, GSDME^T6E^ failed to bind CASP3^C/A^p17-HA/p12 (Fig. 5j, top; Supplementary information, Fig. S5j), and mannose barely influenced the recognition of GSDME^T6A^ by CASP3^C/A^p17-HA/p12 (Fig. 5j, bottom). Circular dichroism (CD) spectrum analysis showed that the conformation of GSDME^T6E^ is different from that of wild-type (WT) GSDME (Supplementary information, Fig. S5k), indicating that Thr6 phosphorylation indeed induces conformational change of GSDME. Together, it could be concluded that mannose-activated AMPK may induce the conformational change of GSDME through phosphorylation of GSDME at Thr6, thereby interfering with the interaction between GSDME and caspase-3.

### Mannose suppresses cisplatin-induced pyroptosis in normal cell lines and organoids

**Fig. 6:**
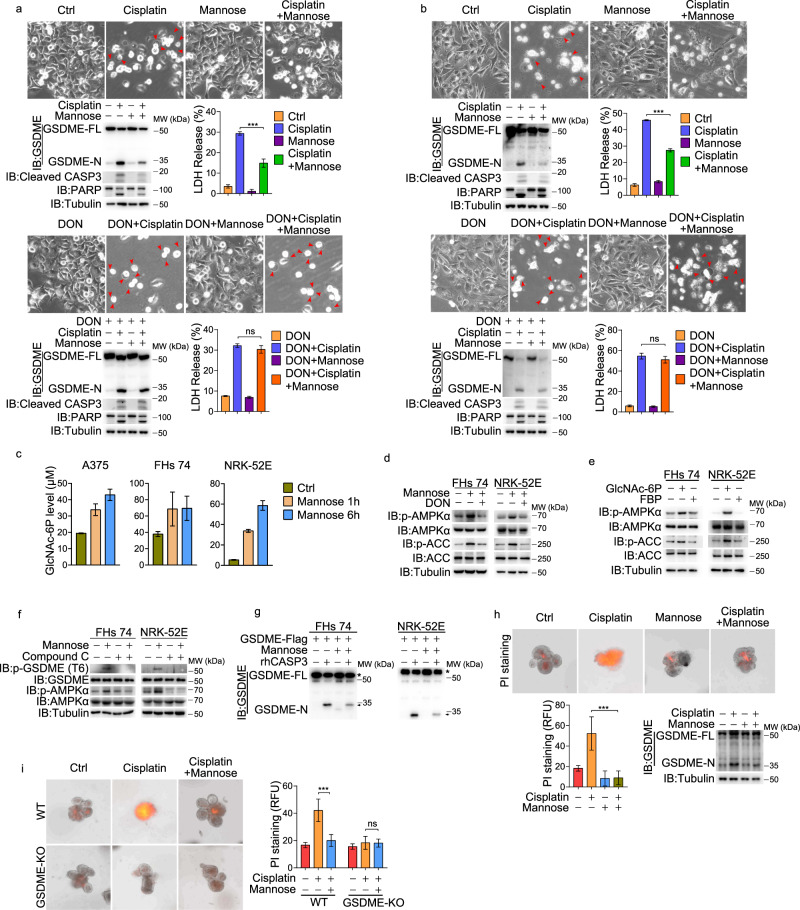
Roles of mannose in cisplatin-induced pyroptosis in normal cell lines and organoids. Normal cell lines were pretreated with mannose (20 mM) for 2 h, and then cisplatin (10 μg/mL) for 24 h to assess pyroptotic features (including characteristic morphology, GSDME cleavage, and LDH release), and the cleaved caspase-3 and its substrate PARP levels were also detected, unless specifically defined. **a**, **b** Top, FHs 74 cells (**a**) and NRK-52E cells (**b**) were pretreated with mannose and then cisplatin. Bottom, cells were pretreated with mannose and DON (40 μM) and then cisplatin. Pyroptosis was determined. **c** The level of GlcNAc-6P in cells detected by LC-MS. **d** Two normal cell lines were treated with mannose with or without DON co-treatment. AMPK and ACC phosphorylation levels were detected. **e** SLO (200 ng/mL) and metabolite GlcNAc-6P (1 mM) were incubated with cells for 10 min. The phosphorylation levels of AMPK and ACC were detected. FBP was used as a negative control. **f** Mannose induced GSDME phosphorylation at T6 site. **g** GSDME was expressed in cells with or without mannose treatment. GSDME proteins were extracted and incubated with reconstituted protein caspase-3. The cleavage of GSDME was detected. The asterisks indicate GSDME-Flag and the arrows indicate cleaved GSDME. **h**, **i** Mannose reversed cisplatin-induced pyroptosis (indicated by GSDME cleavage) and pyroptotic cell death (indicated by PI staining) in small intestinal organoids (**h**) and cisplatin could not induce pyroptotic cell death in GSDME knockout intestinal organoids (**i**). Tubulin was used to determine the amount of loading proteins. All data are presented as mean ± SD of two independent experiments, and one of western blotting results is presented. ***P < 0.001; ns not significant.

The above results demonstrate that mannose is a powerful inhibitor for GSDME-mediated pyroptosis in melanoma cells. It was reported that GSDME-mediated pyroptosis in normal tissues was a major cause of chemotherapy-induced side effects.^15^ Therefore, we tested whether mannose could protect normal tissues from chemotherapy-induced injury caused by pyroptosis. Cisplatin, an agent for chemotherapy to treat various types of cancer in clinics, causes serious side effects, such as gastrointestinal toxicity and nephrotoxicity.^27^ GSDME was expressed at higher levels in the small intestine and kidney of mice (Supplementary information, Fig. S6a); we thus investigated the role that mannose could play in normal intestinal and renal epithelial cells. Indeed, cisplatin clearly induced GSDME-mediated pyroptosis in the human normal intestinal FHs 74 Int (termed FHs 74) cells and rat kidney epithelial NRK-52E cells (Fig. 6a, b, top), and necroptosis was also weakly involved in cisplatin-induced cell death, as revealed by the data that NSA and Nec-1 (inhibitors for necroptosis) showed slight repression on cell death (Supplementary information, Fig. S6c), in line with previous reports.^15,28^ The cisplatin-induced pyroptosis was substantially suppressed by mannose (Fig. 6a, b, top), which was accompanied by the elevation of GlcNAc-6P concentration and AMPK activity (Fig. 6c, d). When cells were co-treated with DON, mannose failed to activate AMPK and suppress cisplatin-induced pyroptosis (Fig. 6a, b, bottom; d), supporting that HBP was required for mannose’s function. Furthermore, the activation of AMPK by GlcNAc-6P (Fig. 6e), the phosphorylation of GSDME Thr6 by mannose-activated AMPK (Fig. 6f), and the suppression of caspase-3-mediated GSDME cleavage by mannose (Fig. 6g) were also observed in these two normal cell lines. With the extension of cisplatin treatment, LDH release and cell death gradually increased, and co-treatment of mannose showed an inhibitory effect (Supplementary information, Fig. S6b). These results all implicate the similar mechanism of mannose function in normal and cancer cells.

To further demonstrate the direct protective effect of mannose in the small intestine, we established small intestinal organoids from GSDME WT and knockout mice (Supplementary information, Fig. S6d). We found that cisplatin clearly induced pyroptosis (propidium iodide (PI) staining; Fig. 6h, top) with the elevation of GSDME cleavage (Fig. 6h, bottom) in the WT small intestinal organoids but not in the GSDME-knockout ones (Fig. 6i), and this cisplatin-induced pyroptosis was dramatically inhibited by mannose (Fig. 6h, i). Observation from videos also showed that small intestinal organoids underwent pyroptosis followed by cell death, and this cell death was inhibited by mannose (Supplementary information, Fig. S6e and Videos S1, S2). In addition, in small intestinal organoids from RIPK3-knockout mice, cisplatin induced cell death and mannose still played an inhibitory role in GSDME cleavage (Supplementary information, Fig. S6f, g). Together, these results demonstrate a protective role of mannose in both normal cells and organoids through blockage of cisplatin-induced pyroptosis.

### Mannose protects organs against chemotherapy-induced injury in mouse models

We further tested the protective role of mannose in mice. When mice were administered with mannose, we detected obvious AMPKα phosphorylation in different tissues such as colon, stomach, liver and kidney, while co-treatment of DON attenuated mannose-induced AMPKα phosphorylation (Supplementary information, Fig. S7a). We established another mouse model, in which C57BL/6 mice were intragastrically administered with mannose one day prior to the peritoneal injection of cisplatin (Supplementary information, Fig. S7b). In the intestine, cisplatin treatment led to extensive colon shortening (Supplementary information, Fig. S7c), loss of crypt’s cylindrical and tightly arranged structures, reduced length and destroyed finger-like structures in villi (Supplementary information, Fig. S7d), and cleavage of GSDME (Supplementary information, Fig. S7e, left). In contrast, co-treatment with mannose effectively protected the small intestine from damage by activating AMPK and suppressing GSDME cleavage (Supplementary information, Fig. S7c–e). Interestingly, this protective effect of mannose in the intestine was also observed in DSS-induced ulcerative colitis, albeit via different mechanisms.^29^ The nephrotoxicity of cisplatin was also inhibited by mannose, as revealed by periodic acid-Schiff (PAS) staining of renal sections (Supplementary information, Fig. S7f, top) and the determination of different indexes, including the levels of blood urea nitrogen (BUN), serum creatinine, and serum cystatin C (Supplementary information, Fig. S7f, bottom); these changes were also accompanied with an increase in AMPK phosphorylation and a decrease in GSDME cleavage in the presence of mannose (Supplementary information, Fig. S7e, right). In colon and kidney, mannose administration also elevated GlcNAc-6P concentration (Supplementary information, Fig. S7g). Clearly, mannose plays a protective role against cisplatin-induced toxicity in the small intestine and kidney, which might be associated with the suppression of pyroptosis by GlcNAc-6P-activated AMPK.

**Fig. 7:**
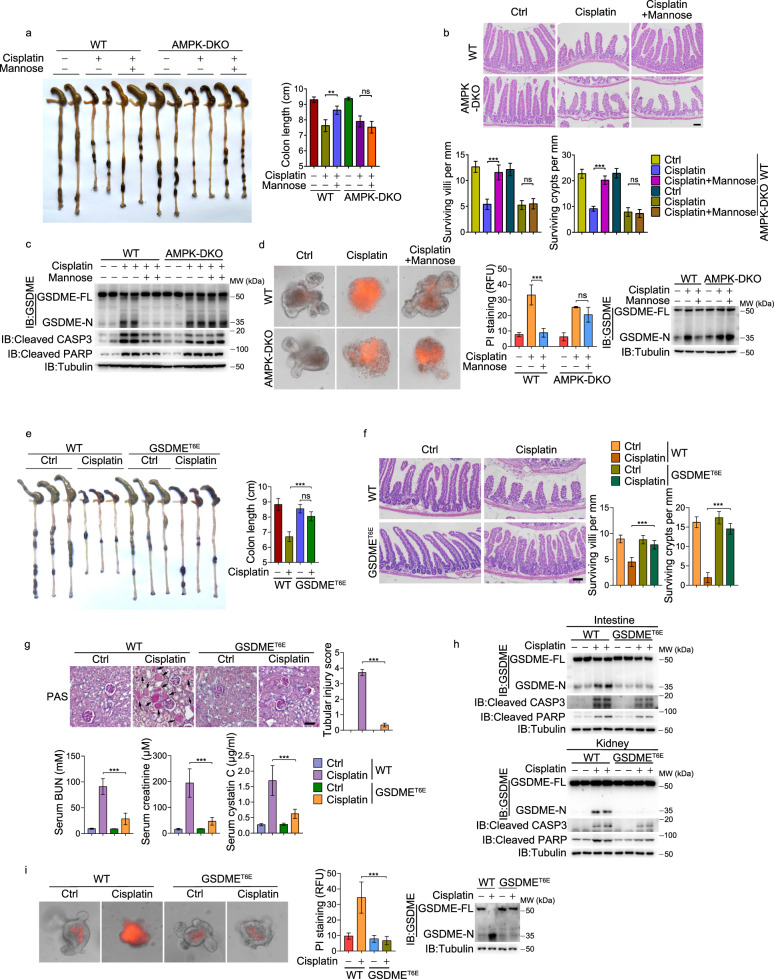
Mannose protected organs against chemotherapy drug-induced injury in mouse models. **a**–**c** In intestine-specific AMPK-DKO mice (*n* = 4), mannose lost its effects on protecting small intestine against cisplatin-induced damages, including colon shortening (**a**), loss of crypts and the reduced villus lengths (**b**; scale bar, 100 μm), and cleavage of GSDME, caspase-3 and its substrate PARP (**c**). **d** Mannose lost its effects on reversing cisplatin-induced pyroptosis (indicated by GSDME cleavage and PI staining) in small intestinal organoids derived from intestine-specific AMPK-DKO mice. **e**–**h** In GSDME^T6E^ knock-in mice (*n* = 6), cisplatin lost its effects on inducing damages of small intestine and kidney, including colon shortening (**e**), loss of crypts and the reduced villus lengths (**f**; scale bar, 100 μm), damages of kidney (**g**, top; scale bar, 100 μm), the increased levels of serum BUN, serum creatinine and serum cystatin C (**g**, bottom), and cleavage of GSDME, caspase-3 and its substrate PARP in both intestine and kidney (**h**). **i** Cisplatin was not able to induce pyroptosis (indicated by GSDME cleavage and PI staining) in small intestinal organoids derived from GSDME^T6E^ knock-in mice. Tubulin was used to determine the amount of loading proteins. All data are presented as mean ± SD of two independent experiments, and one of western blotting results is presented. ***P < 0.001, **P < 0.01; ns not significant.

To further verify the potential role of AMPK in the protection of normal tissues from cisplatin-induced injury, the intestine-specific AMPKα1/α2 double knockout mice (*AMPKα1/α2*^*fl/fl*^;*Villin-Cre*, termed AMPK DKO) were employed. Although the knockout of AMPKα1/α2 did not affect cisplatin-induced intestinal toxicity, it clearly abolished the protective effects of mannose in the cisplatin-induced colon shortening and intestinal villus damage (Fig. 7a, b). Mannose also failed to suppress the cleavage of GSDME in the intestine of AMPK-DKO mice (Fig. 7c). We also generated small intestinal organoids from WT and AMPK-DKO mice (Supplementary information, Fig. S7h). We found that cisplatin was equally effective in inducing GSDME cleavage and pyroptotic cell death in organoids from the AMPK-DKO mice and the WT mice, while mannose basically lost its ability to suppress pyroptosis in AMPK-DKO intestinal organoids (Fig. 7d). These results demonstrated that AMPK plays a crucial role in protecting the intestine from chemotherapy-induced damage.

To further clarify the signaling axis from AMPK to GSDME phosphorylation that suppresses cisplatin-induced pyroptosis in vivo, we separately generated GSDME^T6E^ and GSDME^T6A^ knock-in mice (Supplementary information, Fig. S7i). These strains of mice developed normally, showing no obvious difference in GSDME expression levels in either the small intestine or kidney (Supplementary information, Fig. S7j). The GSDME^T6E^ mice could tolerate cisplatin-induced tissue toxicity well, as revealed by the observation that cisplatin hardly induced any colon shortening (Fig. 7e), villus and crypt disruption (Fig. 7f), and the increase in indexes indicative of kidney injury (Fig. 7g). These phenotypes were closely associated with the failure of GSDME^T6E^ cleavage upon cisplatin administration (Fig. 7h). As expected, small intestinal organoids from the GSDME^T6E^ knock-in mice showed no response to cisplatin as compared to those from WT mice (Fig. 7i). By contrast, in GSDME^T6A^ knock-in mice, cisplatin efficiently induced damages of the intestine and kidney, which could not be relieved by mannose (Supplementary information, Fig. S7k–n). These data strongly demonstrated that mannose protects against the side effects of cisplatin through AMPK-induced GSDME phosphorylation at Thr6.

### The combination of mannose with chemotherapy drugs in the treatment of primary tumors in mouse and clinical patients

**Fig. 8:**
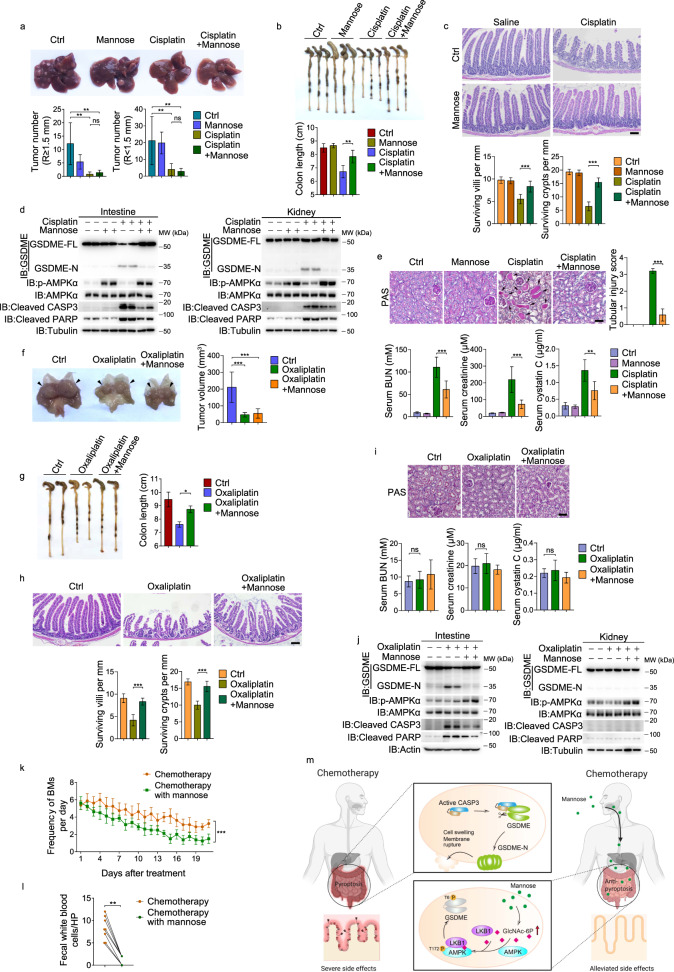
Mannose exerts a protective role in small intestine and kidney in mice and patients. **a**–**e** DEN/CCl_4_-inducd HCC mice (*n* = 5) were treated with cisplatin in the absence or presence of mannose. In liver, the numbers of tumor were counted (**a**). Mannose rescued cisplatin-induced damages of intestine, including colon shortening (**b**), loss of crypts and the reduced villus lengths (**c**; scale bar, 100 μm). Mannose inhibited cisplatin-induced cleavage of GSDME, caspase-3 and its substrate PARP, while elevated AMPK phosphorylation in intestine and kidney (**d**). Mannose rescued cisplatin-induced damages of kidney, including decreases in the tubular injury score (**e**, top; scale bar, 100 μm), the levels of serum BUN, serum creatinine, and serum cystatin C (**e**, bottom). **f**–**j** Transgenic mice with gastric mucosa-specific expression of COX-2/mPGES-1 (*n* = 3) were treated with oxaliplatin in the absence or presence of mannose. In hyperplastic stomach, tumor volumes were counted (**f**). Mannose rescued oxaliplatin-induced damages of intestine, including colon shortening (**g**), loss of crypts and the reduced villus lengths (**h**; scale bar, 100 μm). Oxaliplatin induced less damages in kidney (**i**; scale bar, 100 μm). Mannose inhibited oxaliplatin-induced cleavage of GSDME, caspase-3 and its substrate PARP, while elevated AMPK phosphorylation detected in small intestine and kidney (**j**). **k**, **l** The eight patients received normal chemotherapy with XELOX regimen with or without mannose supplement. Numbers of BMs were recorded daily (**k**) and fecal WBCs were shown at the end of chemotherapy cycle (**l**). **m** A working model for functional mechanism of mannose. Once entering cells, mannose elevates metabolite GlcNAc-6P level, which facilitates interaction of cytosol AMPK and LKB1, leading to AMPK phosphorylation. Activated AMPK phosphorylates GSDME at T6, and results in the failure of GSDME cleavage by caspase-3, thereby inhibiting pyroptotic occurrence. Tubulin or actin was used to determine the amount of loading proteins. All data are presented as mean ± SD of two independent experiments, and one of western blotting results is presented. ***P < 0.001, **P < 0.01, *P < 0.05; ns not significant.

The above results strongly suggest that cisplatin-induced damages in normal organs can be protected by mannose and a combined use of mannose and chemotherapeutic agents may achieve the best result for tumor therapy with the induction of apoptosis in primary tumors by chemotherapeutic agents while inhibiting the chemotherapy-induced pyroptosis in normal tissues by mannose. To test this, the potential application of mannose in ameliorating cisplatin-induced tissue damage in the diethylnitrosamine (DEN)/carbon tetrachloride (CCl_4_)-induced primary liver tumor model was investigated (Supplementary information, Fig. S8a).^30^ Cisplatin administration obviously retarded liver tumor progression (Fig. 8a) through inducing apoptosis in liver tumor (Supplementary information, Fig. S8b), but also induced acute injuries in the intestinal tract and kidney, such as colon shortening, destruction of the crypt and villus (Fig. 8b, c), and increase of the indexes for kidney injury (Fig. 8e). These cisplatin-induced injuries were closely associated with the cleavage of GSDME in intestine and kidney tissues (Fig. 8d). Co-treatment of mannose did not dampen the cisplatin-induced apoptosis in tumor tissues (Supplementary information, Fig. S8b), but effectively ameliorated cisplatin-induced injuries in intestine and kidney with the activation of AMPK (Fig. 8b–e). Therefore, mannose effectively alleviates cisplatin-induced nephrotoxicity and gastrointestinal toxicity without affecting the pro-apoptotic effect of cisplatin in primary tumors.

We further used transgenic mice with gastric mucosa-specific expression of cyclooxygenase 2 (COX-2) and microsomal prostaglandin E synthase (mPGES)-1 (termed K19-C2mE), which develop primary hyperplastic gastric tumors,^31^ to test another first-line chemotherapeutic agent, oxaliplatin (Supplementary information, Fig. S8c).^32^ Oxaliplatin could effectively inhibit gastric tumor growth (Fig. 8f) through inducing apoptosis (Supplementary information, Fig. S8d), and the oxaliplatin-induced side effects mainly occurred in the intestine (Fig. 8g, h) but not in the kidney (as oxaliplatin has few side effects on the kidney^33^) (Fig. 8i), probably owing to the activation of GSDME cleavage in the small intestine but not in the kidney (Fig. 8j). Mannose also had a protective effect in the small intestine through inhibiting GSDME cleavage without impairment of oxaliplatin-induced apoptosis in primary tumors (Fig. 8f–j; Supplementary information, Fig. S8d).

Recently, it was reported that mannose enhances chemotherapy by activating apoptosis in tumor cells, which is dependent on the low expression of PMI.^20^ By contrast, high PMI expression is required for the protective effects of mannose in pyroptosis, as revealed by the results that PMI was highly expressed in different cancer or normal cell lines we used (Supplementary information, Fig. S8e), and knockdown of PMI in high PMI-expressing cells not only abolished the protective effect of mannose, but also even mildly promoted cell death induced by different drugs (Supplementary information, Fig. S8f). Similarly, PMI was also highly expressed in both DEN/CCl_4_-induced liver tumors and K19-C2mE gastric tumors (Supplementary information, Fig. S8g, top), and small intestine and kidney tissues in normal mice (Supplementary information, Fig. S8g, bottom). Therefore, our results not only support that low PMI expression is required for mannose to promote apoptosis,^20^ but also clarifies that high PMI expression dictates the anti-pyroptotic effect of mannose. In addition, mannose did not obviously affect the blood glucose level in mice (Supplementary information, Fig. S8h). Oral administration of mannose in mice increased the plasma levels of mannose (Supplementary information, Fig. S8i).

We also detected the activation of caspase-3 upon mannose treatment. Unexpectedly, mannose treatment not only suppressed GSDME cleavage, but also decreased the activity of caspase-3 upon the stimulation of CCCP/iron or raptinal (Figs. 1b, e–g, 5h). Similarly, in normal cells or tissues, cisplatin-activated caspase-3 activity was also inhibited by mannose (Figs. 6a, b, 7c, h, 8d, j; Supplementary information, Fig. S7e, m). Given that we did not detect any association of AMPKα1 with caspase-3 (Supplementary information, Fig. S5a), and that N-terminus of GSDME was reported to function as an upstream factor for caspase-3 activation through inducing permeabilization of mitochondrial outer membrane and the release of cytochrome c from mitochondria,^34^ we conjectured that the decrease of caspase-3 activity by mannose may be due to the inhibition of GSDME cleavage. To test this, we knocked down GSDME in cells and found that mannose could no longer inhibit CCCP/iron-induced activation of caspase-3 and release of cytochrome c from mitochondria (Supplementary information, Fig. S8j, k). Similarly, in GSDME-null PC-9 cells and MDA-MB-468 cells, only when GSDME was ectopically overexpressed, mannose could impair raptinal-induced caspase-3 activation (Fig. 1f, g). Furthermore, when WT GSDME or GSDME^T6A^ were overexpressed in GSDME-knockout A375 cells, mannose inhibited the CCCP/iron-induced caspase-3 activation in WT GSDME- but not GSDME^T6A^-expressing cells (Fig. 5h). Transfection of GSDME, but not GSDME^T6E^, into GSDME-knockout A375 cells enhanced the activity of caspase-3 upon CCCP/iron stimulation (Fig. 5g). In mouse models, knockout of GSDME impaired the activation of caspase-3 by cisplatin in the kidney and intestine (Supplementary information, Fig. S8l). Mannose also decreased cisplatin-induced caspase-3 activity in the intestine and kidney from WT mice but not GSDME^T6A^ knock-in mice (Supplementary information, Figs. S7e, m, 8d, j (for Oxaliplatin)). Together, this series of results consistently demonstrate that although GSDME is a substrate of caspase-3, cleaved GSDME also functions as an upstream factor for caspase-3 activation as reported previously.^34^ Therefore, mannose suppresses GSDME cleavage to interrupt this feedback loop in caspase-3-GSDME axis, thereby inhibiting caspase-3 activation.

The results from mouse models suggest that induction of apoptotic cell death in tumors by chemotherapy while suppression of pyroptotic cell death in normal organs by mannose may represent a practicable strategy for clinical cancer therapy. XELOX regimen (capecitabine plus oxaliplatin, 21 days per chemotherapy cycle) usually causes gastrointestinal toxicity in patients, such as a high diarrhea incidence.^35^ To evaluate the effectiveness of mannose in alleviating chemotherapy-induced side effects, eight gastrointestinal cancer patients receiving XELOX regimen were recruited. Patients received conventional XELOX regimen (as a control cycle) suffered from severe diarrhea, and bowel movements (BMs) occurred 5–6 times per day in the first 5 days and even 3–4 times per day in the last 5 days of the chemotherapy cycle. However, in the sequential chemotherapy cycle, the same patients received XELOX regimen plus mannose obviously decreased the frequency of daily BMs, i.e., 4–5 times per day in the first 5 days and 1–2 times per day in the last 5 days (Fig. 8k, Table 2; Supplementary information, Table S2). The average number of fecal WBCs from these 8 patients in the control cycle was 8.25 per field, which was effectively decreased to 1.25 per field in the sequential chemotherapy cycle by mannose supplement (Fig. 8l, Table 3). These results suggested the efficacy of mannose in alleviating chemotherapy-induced intestinal toxicity. In addition, biochemical markers, such as alanine transaminase (ALT), Aspartate transferase (AST), serum creatinine, BUN and WBC detected in the blood remained stable within the normal range after mannose supplement (Table 3; Supplementary information, Fig. S8m, Table S2). Clinical observation showed that this reduction of side effects could greatly restore the vitality of the patients. Therefore, it illustrates the prospect of mannose as an auxiliary drug for clinical chemotherapy to alleviate side effects in patients.

**Table 2:** Effect of mannose on XELOX-induced diarrhea.

Frequency of daily BMs	without mannose (*n* = 8), mean (95% CI)	with mannose (*n* = 8), mean (95% CI)	*P* value
Complete cycle (d1–d21)	4.33 (4.14–4.51)	2.94 (2.72–3.15)	< 0.001
d1–d5	5.63 (5.37–5.88)	4.83 (4.56–5.10)	0.002
d6–d10	4.75 (4.46–5.03)	3.40 (3.20–3.60)	< 0.001
d11–d16	3.96 (3.74–4.17)	2.21 (2.02–2.40)	< 0.001
d17–d21	3.05 (2.86–3.24)	1.45 (1.29–1.61)	< 0.001

**Table 3:** Effect of mannose on XELOX-induced side effects.

Characteristic	without mannose (*n* = 8), mean (95% CI)	with mannose (*n* = 8), mean (95% CI)	*P* value
Fecal WBC (/HP)	8.25 (6.07–10.43)	1.25 (0.38–2.12)	0.008
Blood WBC (10^9^/L)	7.19 (6.27–8.12)	6.70 (5.92–7.48)	0.202
Serum ALT (U/L)	37.44 (31.21–43.67)	37.1 (31.01–43.19)	0.690
Serum AST (U/L)	28.16 (22.82–33.50)	29.61 (23.56–35.67)	0.111
Serum BUN (mM)	5.74 (4.78–6.69)	5.71 (4.78–6.64)	0.769
Serum creatinine (μM)	75.15 (67.90–82.40)	73.63 (68.09–79.16)	0.575

## Discussion

Pyroptosis is a type of lytic cell death mediated by gasdermin family members. However, how gasdermin-mediated pyroptosis can be negatively regulated is largely unknown. Herein, we demonstrated a unique mannose-induced signaling pathway associated with AMPK activation by the metabolite GlcNAc-6P to phosphorylate GSDME, which leads to the failure of caspase-3 to cleave GSDME, thereby reversing pyroptosis. Furthermore, this mannose-induced pyroptotic repression exhibits important physiological significance in clinical chemotherapy, as mannose has the ability to protect normal organs from chemotherapeutic drug-induced injury, as verified in several mouse models and patients (Fig. 8m).

Chemotherapeutic agents such as platinum-based drugs are regularly prescribed for cancer treatment. Although they are effective in suppressing many types of cancer, their usage is largely limited owing to severe side effects. For platinum drugs, nephrotoxicity, gastrointestinal toxicity, neurotoxicity and ototoxicity are commonly described side effects.^27^ The finding that GSDME was highly expressed in kidney, small intestine, brain and cochlea, as revealed by this study and other reports,^36^ supports the notion that uncontrolled GSDME-mediated pyroptosis in normal organs is one of the major causes of the side effects induced by chemotherapy.^15^ Therefore, achieving the selective induction of cell death in tumors (such as apoptosis) and suppression of GSDME-mediated pyroptosis in normal organs is an arduous and long-term challenge for clinical cancer therapy. Our data demonstrated that mannose represents an ideal complementary agent for chemotherapy, as it effectively managed side effects without impairing the antitumor activity of the chemotherapeutic agents, cisplatin and oxaliplatin. Mannose is a naturally occurring C-2 epimer of glucose and is widely used as a dietary supplement. The physiological level of mannose in human blood is ~100 µM,^37^ and humans can tolerate supraphysiological doses of mannose (up to 2 mM in the blood) without signs of liver or renal toxicity,^38^ demonstrating the safety of mannose administration. The clinical application of mannose in the treatment of urinary tract infections^39^ and congenital disorders of glycosylation type Ib^40^ has been reported. Previous studies have also indicated that mannose can suppress tumor growth and enhance chemotherapy.^20^ Our study further extends the benefits of mannose to the alleviation of chemotherapy-induced side effects by the inhibition of GSDME-mediated pyroptosis in normal organs, but mannose did not dampen apoptosis in tumor tissues. Moreover, given the proinflammatory nature of pyroptosis and the anti-inflammatory properties of mannose,^29,41^ mannose may possess broader application prospects in managing the chemotherapy-induced side effects through a multipronged approach and could potentially be used clinically in cancer chemotherapy.

The benefit of taking mannose for tumor patients who receive chemotherapy was further verified. Diarrhea, which is a sign of gastrointestinal toxicity in patients, is a common adverse event for chemotherapy.^42^ Eight patients diagnosed with gastrointestinal tumor and suffered from severe diarrhea during postoperative adjuvant chemotherapy (XELOX regimen), were enrolled in our study. Mannose effectively alleviated the frequency of diarrhea in patients, and to some extent alleviated the suffering brought to patients by chemotherapy. By contrast, loperamide, a regularly prescribed drug in clinic against diarrhea through decreasing gastrointestinal motility, has no effect in alleviating chemotherapy-induced damage in intestines.^43^ Hence, mannose may possess systemic benefit for tumor patients receiving chemotherapy through inhibiting pyroptosis in normal organs. Since the indexes for renal injury in the end of chemotherapy cycle were within the normal range in this pilot clinical study, we could not evaluate the protective effect of mannose in kidney. The protective effect of mannose in multiple tissues should be further verified by more comprehensive clinical trials with larger cohort in the future.

Our study provides evidence that the antipyroptotic effect of mannose can be attributed to the activation of AMPK, which phosphorylates GSDME at Thr6 to suppress GSDME-mediated pyroptosis in normal organs. Knockout of AMPK in mice abolished the protective effect of mannose against tissue toxicity. It has been reported that Thr6 phosphorylation prevents the oligomerization of GSDME-N, thereby inhibiting the pore-forming activity of GSDME;^34^ however, the kinase involved in Thr6 phosphorylation is still unknown. Our study has not only clarified the role of AMPK as the kinase responsible for the phosphorylation of GSDME at Thr6, but also demonstrated that Thr6 phosphorylation inhibits the recognition of GSDME by caspase-3 to suppress GSDME cleavage, indicating novel crosstalk in AMPK-mediated GSDME Thr6 phosphorylation. These findings thus explain why metformin, a commonly used hypoglycemic drug that activates AMPK, could also alleviate chemotherapy-induced tissue injury in mice.^44^ In this regard, it will be interesting to evaluate whether diabetic patients who take metformin would be more tolerant of chemotherapy-induced side effects. Notably, the Ser/Thr residue that corresponds to Thr6 in GSDME is also present in GSDMA (Thr8), GSDMB (Thr9) and GSDMC (Ser9) but not in GSDMD, implying that phosphorylation at these sites may also play a role in the negative regulation of GSDMA-, GSDMB- or GSDMC-mediated pyroptosis. However, we did not observe any inhibitory effect of mannose on GSDMC-dependent pyroptosis, which suggests that either other kinases may be responsible for the phosphorylation of GSDMC Ser9 or AMPK-induced Thr6 phosphorylation specifically affects the recognition of only GSDME (but not other gasdermins) by only caspase-3 (but not other proteases). In addition, GSDMC is cleaved by caspase-8,^23,45^ but whether pyroptosis mediated by high GSDMC expression is also responsible for normal tissue injury is still unknown. These are interesting issues that deserve further study.

AMPK, an energy-sensing protein kinase, is activated by increasing AMP/ATP and ADP/ATP ratios.^46^ Recently, it was reported that AMPK can also sense glucose availability, as a decrease in the glycolytic intermediate FBP leads to the assembly of the Axin-based AMPK activation complex on the surface of lysosomes to activate AMPK.^5,46,47^ Mannose treatment did not influence the AMP/ATP ratio or ADP/ATP ratio, in line with a previous report.^20^ Although it was suggested that a decrease in FBP levels may contribute to mannose-activated AMPK,^20^ the exact mechanism underlying mannose-induced AMPK activation is still unknown. Within cells, the metabolism of mannose is associated with three different pathways: the glycolysis pathway, HBP, and the GDP-mannose biosynthesis pathway. We verified that the flow of mannose metabolism toward HBP is essential for AMPK activation, as genetic depletion or pharmacological inhibition of GFAT, a rate-limiting enzyme in the HBP, completely blocked mannose-induced AMPK activation. Among the intermediates of the HBP, both GlcNAc-6P and GlcNAc-1P were able to activate AMPK in a permeabilized cell system, but only GlcNAc-6P was elevated upon mannose stimulation, and GlcNAc-6P is thus involved in mannose-induced AMPK activation in cells. GlcNAc-6P directly binds the AMPKα subunit to facilitate the recognition and phosphorylation of AMPKα by its upstream kinase LKB1. Hence, it is likely that GlcNAc-6P functions as a naturally occurring allosteric metabolite to activate AMPK. Although the physiological relevance of AMPK-mediated GlcNAc-6P sensing is elusive, cells may take advantage of this mechanism to maintain the homeostasis of HBP, which plays critical roles in nutrient sensing.^26,48^ The accumulation of GlcNAc-6P activates AMPK, and activated AMPK can then phosphorylate GFAT to impair GFAT activity,^49,50^ thereby diminishing the further flow of metabolites into HBP. Pathologically, excessive activation of HBP, which is common in cancer,^51,52^ may partially endow cancer cells with the ability to escape GSDME-dependent pyroptosis by activating AMPK. Clearly, it will be important to interpret the structural basis of this AMPK activation by GlcNAc-6P in the future.

## Materials and methods

### Reagents and antibodies

Chemical reagents: CCCP (Cat# C2759), raptinal (Cat# SML1745), dimethyl α-ketoglutarate (Cat# 349631), glucosamine-6P (Cat# G5509), GlcNAc-1P (Cat# A2142), UDP-GlcNAc (Cat# U4375) and *N*-acetyl-L-cysteine (NAC) (Cat# A9165) were purchased from Sigma-Aldrich. Mannose (Cat# A600554), glucose (Cat# A600219), PI (Cat# A601112), HEPES (Cat# A600264) and gentamycin sulfate (Cat# A506614) were purchased from Sangon Biotech. FeSO_4_ (Cat# 10012118) and CCl_4_ (Cat# 10006418) were purchased from Sinopharm. Cisplatin (Cat# HY-17394), lipopolysaccharides (Cat# HY-D1056), 3× FLAG peptide (Cat# HY-P0319A), Y-27632 (Cat# HY-10583), CQ (Cat# HY-17589A), Ferrostatin-1 (Cat# HY-100579), MG132 (Cat# HY-13259) and metformin (Cat# HY-17471A) were purchased from MedChemExpress. Liproxstatin-1 (Cat# S7699) was purchased from Selleck. Fructose (Cat# F108335), F6P (Cat# F113774), FBP (Cat# F111301), fucose (Cat# F100396), GlcNAc (Cat# A118965), GlcN (Cat# G119456) and *N*-nitrosodiethylamine (Cat# N109571) were purchased from Aladdin. Human TNF-α protein (Cat# 10602-HNAE) and recombinant murine EGF (Cat# 10605-HNAE) were purchased from Sino Biological. Protease inhibitor cocktail (Cat# K1007), phosphatase inhibitor cocktail (Cat# K1015), nigericin (Cat# B7644), DON (Cat# c3575), Z-VAD-FMK (Cat# A1902), and compound C (Cat# B3252) were purchased from ApexBio. Cycloheximide (Cat# GC17198) was purchased from GlpBio. MMAE (Cat# T6897) was purchased from Topscience. Advanced DMEM/F12 (Cat# 12491015), B-27™ supplement (Cat# 17504044) and N2 supplement (Cat# 17502048) were purchased from Gibco. MK-8722 (Cat# M424840) was purchased from Toronto Research Chemicals. GlcNAc-6P (Cat# 295202) was purchased from J&K Scientific. SLO (Cat# S4470) and insulin (Cat# I8040) were purchased from Solarbio. Recombinant human caspase-3 protein (Cat# 707-C3-010/CF) was purchased from R&D Systems. GlutaMAX™ supplement (Cat# 35050061) was purchased from Thermo Fisher Scientific. GFR basement membrane matrix (Cat# 356231) was purchased from Biocoat. A nonessential amino acid (NEAA) solution (Cat# S220JV) was purchased from BasalMedia.

Antibody: anti-GSDME (Cat# ab215191) and anti-GSDMD (Cat# ab210070) antibodies were purchased from Abcam. Anti-GSDME for immunoprecipitation (IP) (Cat# 13075-1-AP), anti-GFAT1 (Cat# 14132-1-AP), anti-GFAT2 (Cat# 15189-1-AP), anti-PFKP (Cat# 13389-1-AP), anti-PFKM (Cat# 55028-1-AP), anti-GNPNAT1 (Cat# 16282-1-AP), anti-GPI (Cat# 15171-1-AP), anti-UAP1 (Cat# 67545-1-Ig), anti-ACC1 (Cat# 21923-1-AP), anti-CaMKK2 (Cat# 11549-1-AP), anti-Myc (Cat# 60003-2-Ig), anti-cleaved PARP (Cat# 13371-1-AP) and anti-actin (Cat# 66009-1-Ig) antibodies were purchased from Proteintech. Anti-phospho-AMPKα (Cat# 2535S), anti-AMPKα (Cat# 2603S), anti-phospho-ACC (Cat# 3661S), anti-cleaved caspase-3 (Cat# 9661S), anti-PARP (Cat# 9532) and anti-LKB1 (Cat# 3050S) antibodies were purchased from Cell Signaling Technology. Anti-GSDMC (Cat# A14550), anti-PMI (Cat# A7319) and anti-PGM3 (Cat# A15698) antibodies were purchased from ABclonal. Anti-phosphoserine/threonine (Cat# 612549) antibody was purchased from BD Transduction Laboratories™. Anti-HA (Cat# H-9658), anti-Flag (Cat# F-1804) and anti-tubulin (Cat# T-4026) antibodies were purchased from Sigma-Aldrich. Goat anti-rabbit (Cat# 31210) and anti-mouse (Cat# 31160) secondary antibodies were purchased from Thermo Fisher Scientific.

### Cell culture

The human embryonic kidney cell line HEK293T, the melanoma cell lines A375, IgR3, M14, MEL-RM, SK-MEL-1, the cervical cancer cell line HeLa, colon carcinoma cell line RKO and the rat kidney epithelial cell line NRK-52E were cultured in Dulbecco’s modified Eagle’s medium (DMEM, Sigma). Human small intestine epithelial FHs 74 cells were cultured in DMEM supplemented with 10 μg/mL insulin. Human monocyte-like cell line THP-1, human bone osteosarcoma epithelial cell line U2OS and human lung cancer cell line PC-9 were cultured in RPMI 1640 medium. The human osteosarcoma cell line SAOS-2 was cultured in McCoy’s 5 A medium. The human breast cancer cell line MDA-MB-468 was cultured in Leibovitz’s L-15 medium and grown in an environment without CO_2_ equilibration. All media were supplemented with 10% fetal bovine serum (FBS), 100 IU penicillin (Cat# A600135, Sangon Biotech), and 100 mg/mL streptomycin (Cat# A610494, Sangon Biotech). PC-9, MDA-MB-468, U2OS, SAOS-2, RKO cell lines were purchased from Xiamen Immocell Biotechnology Co., Ltd. Cells were routinely tested to be negative for mycoplasma.

### Mouse models

All mice were maintained on a 12 h light/12 h dark cycle with free access to food and water. All the animal experiments below were approved by the Animal Ethics Committee of Xiamen University (acceptance No. XMULAC20190048).

For mannose treatment, 6–8-week-old C57BL/6 mice received 20% (w/v) mannose by oral gavage (500 μL) one time daily. Cisplatin was injected intraperitoneally at a concentration of 18 mg/kg. Four days after cisplatin treatment, the mice were euthanized, and the small intestine and kidney were collected for analysis.

For the DEN/CCl_4_-induced hepatocarcinoma model, 15-day-old male mice were intraperitoneally injected with DEN dissolved in PBS at a dose of 25 mg/kg. One week later, the mice were intraperitoneally injected with 10% CCl_4_ (in 0.5 mL of corn oil per kg body weight) weekly for 4–5 months. Then, the mice received 20% (w/v) mannose by oral gavage (500 μL) one time daily. Cisplatin (8 mg/kg) was injected intraperitoneally one time weekly for one month. The mice were euthanized, and the small intestine and kidney were collected for analysis.

GSDME^T6E^ and GSDME^T6A^ mice were constructed by the Xiamen University Laboratory Animal Center. *AMPKα1/α2*^*fl/fl*^*;Villin-Cre* mice were a generous gift from Prof. Sheng-Cai Lin (Xiamen University). GSDME-knockout mice were purchased from GemPharmatech Co., Ltd. RIPK3-knockout mice were purchased from Shanghai Biomodel Organism Science & Technology Development Co., Ltd. The GSDME^T6E^, GSDME^T6A^ or *AMPKα1/α2*^*fl/fl*^*;Villin-Cre* mice were treated with mannose (20%, 500 μL, daily oral gavage for 5 days) and cisplatin (18 mg/kg, intraperitoneally on day 1).

Transgenic mice with gastric mucosa-specific expression of COX-2/mPGES-1 were a generous gift from Prof. Makoto M. Taketo (Kyoto University), and the mice developed obvious hyperplastic gastric tumors after 20 weeks of age.^31^ Forty-eight-week-old COX-2/mPGES-1 transgenic mice were treated with mannose (20%, 500 μL, daily oral gavage for 26 days) and oxaliplatin (3 mg/kg, once a day on days 1–5, days 11–15 and days 21–25).

To obtain small intestine, kidney or other tissue and organ samples, mice were sacrificed, and samples were gently collected and washed with ice-cold 1× PBS and then homogenized with a homogenizer in 2× SDS loading buffer. The samples were boiled for 15 min before being subjected to western blotting.

### Patients and study design

Eight patients diagnosed with gastrointestinal cancer and suffering from severe diarrhea during postoperative adjuvant chemotherapy (XELOX regimen), were enrolled in this study between April 2022 and September 2022 from the First Affiliated Hospital of Harbin Medical University. Written informed consent was obtained from all patients. This study was approved by the First Affiliated Hospital of Harbin Medical University Ethics Committee Board (2022167), and was conducted in accordance with the Helsinki Declaration. Patients between 18 and 65 years old with positive history of chemotherapy-induced diarrhea after receiving XELOX regimen were included. Patients were excluded if they received targeted therapy or immunotherapy, changed chemotherapy regimen, failed to complete the course of chemotherapy. Eligible patients were enrolled during two random but sequential XELOX regimen cycles (21 days per cycle). In the former cycle, patients received conventional XELOX regimen (as controls); in the latter cycle, the same patients were orally given 500 mg D-Mannose (Now Foods Company, USA) once per day from day 1 receiving XELOX regimen and until day 21 when the chemotherapy cycle was completed. Numbers of BMs were recorded per day in these sequential chemotherapy cycles. Patient bloods and feces were collected and evaluated on day 21.

### Plasmid construction

Flag-AMPKα1, HA-AMPKβ1, and Myc-AMPKγ1 were previously established.^53^ The cDNA sequences of GSDME fused with a C-terminal Flag or HA were inserted into pLenti (Addgene 22255). cDNA sequences for AMPKα1 fused with an N-terminal Flag or HA were inserted into pLenti. GSDME-Flag (T6E, T6A, S111E, S424E, S464E) sequences were generated using the QuikChange method and verified by sequencing. cDNA sequences for full-length caspase-3 and fragments p17 and p12 were inserted into pCMV5. GST-AMPKα1, GST-LKB1 and GST-GSDME were constructed using pGEX-4T-1 vectors. His-AMPKα1 was constructed using pET28a vectors. All plasmids were verified by sequencing and purified by TIANprep Midi Plasmid Kit (Tiangen, Cat# DP106). Guide RNAs (gRNAs) were cloned into the lentiGuide-Puro vector (Addgene 52963) as previously described. The sequences of gRNAs are as follows:

sgNTC: AAATGTCAGGCCGCGCCGTT

sgGSDME: AGTCTTCATTTGGAACCCTG

sgGFAT1: CTCCGTACACCAATCAACAG

sgGFAT2: TTCAAGAGTGTCCACTACCC

sgGNPNAT1: TTTAAGTCAGCAGTACAAAG

sgPGM3: ATGGTGTATTGTCGAAACAC

sgUAP1: GCACTGCCTAATACCTCTCG

sgGPI: CCGGTCAAACACACCCATCC

sgPFKP: GAACGCTGCCGTCCGTGCCG

sgPFKM: ATGAAACTCGTCTTTATGCA

sgPMM2: ATCAAAATCGGAGTGGTAGG

sgAMPKα1: TCCTGTTACAGATTGTATGC

sgAMPKα2: ACGTTATTTAAGAAGATCCG

sgLKB1: GTTGCGAAGGATCCCCAACG

sgCaMKK2: ACGTGGTGAGACTCCACTGT

Short hairpin RNA (shRNA) was cloned into the PLKO.1 vector; the sequence of shRNA is as follows:

PMI-shRNA: GCCAGTTGAGGAGATTGTAAC

### Lentivirus production and generation of CRISPR/Cas9 knockout cell lines

Lentiviruses were produced by transfecting HEK293T packaging cells with lentiviral backbone constructs, the packaging plasmid psPAX2 (Addgene 12260) and the envelope plasmid pMD2.G (Addgene 14887) (4 μg:3 μg:1 μg for 6-cm dishes) using the calcium phosphate transfection method. After transfection for 8–12 h, the medium was removed, and fresh warm medium containing NEAA was added. Lentiviral supernatants were collected 48 h after transfection. Target cells were transduced with lentiviruses, and 10 μg/mL polybrene (Sigma, Cat# H9268) was added. After 24 h, the cells were treated with puromycin (InvivoGen, Cat# ant-pr-1) for 2–3 days. Knockout efficiencies were determined by western blotting. The puromycin-resistant cell pools with higher knockout efficiencies (> 80%) were maintained for further experiments.

### Western blot analysis

The cells were lysed in ELB lysis buffer (150 mM NaCl, 100 mM NaF, 50 mM Tris-HCl, pH 7.6, 0.5% Nonidet P-40 (NP-40) containing protease inhibitor cocktail and phosphatase inhibitor cocktail). Cell lysates were centrifuged at 14,000× *g* and 4 °C for 15 min. The supernatants were mixed with an equal volume of 2× SDS loading buffer and boiled for 15 min. The samples were separated by SDS-PAGE, transferred to a PVDF membrane and then analyzed by immunoblotting with the specific antibodies.

### IP analysis

IP was carried out as described previously.^54^ Briefly, cells were lysed in ELB lysis buffer (150 mM NaCl, 100 mM NaF, 50 mM Tris-HCl, pH 7.6, 0.5% NP-40 containing protease inhibitor cocktail and phosphatase inhibitor cocktail). Cell lysates were incubated with specific antibody and protein G-Sepharose beads (Millipore, Cat# 16-266) at 4 °C for 3 h. The immunoprecipitants were collected and washed three times with ELB buffer. For co-IP assay of endogenous proteins, cell lysates were incubated with anti-GSDME antibody (Proteintech) at 4 °C overnight, and then protein G-Sepharose beads were added at 4 °C for 30 min. The immunoprecipitates were mixed with 1× SDS loading buffer. For co-IP analysis of caspase-3 and GSDME, caspase-3(p17, C163A)-HA and caspase-3(p12) were used to transfect A375 cells.

### TurboID assay

TurboID assay was carried out as described previously.^55^ Briefly, culture medium was changed to serum-free culture medium for 0.5 h and 100 μM biotin was added for another 10 min at 37 °C. Then culture medium was further changed to culture medium containing 10% FBS for 2 h and biotin-labeled proteins were purified by using Streptavidin magnetic beads (Thermo Fisher Scientific, Cat# 88816). The samples were prepared for western blot and mass spectrometry analyses.

### GST pull-down assay

GST- or His-tagged proteins were expressed in *E. coli* strain BL21 (DE3) and purified by using glutathione agarose (Thermo Fisher Scientific, Cat# 16101) or HIS-Select® nickel affinity gel (Sigma, Cat# p6611). Bead-bound GST (1 μg) or GST-fusion proteins (1 μg) were incubated with His-tagged proteins (2 μg/mL) in 500 mL of ELB buffer at 4 °C for 2 h. Unbound proteins were removed by washing the beads with ELB buffer three times. The samples were prepared by boiling the mixtures in 1× SDS loading buffer for 10 min.

### Hematoxylin and eosin (H&E) staining of the small intestine

The obtained small intestine samples were washed with PBS until the intestinal lumen was clear. The small intestine was prepared with the Swiss roll technique,^56^ fixed in a solution of 4% formaldehyde and embedded in paraffin. Paraffin sections (5 μm) were dried at 65 °C for 2 h. The tissues were deparaffinized using 100% xylene and 100%, 90%, 75%, and 50% ethanol and then stained with H&E. For immunohistochemical staining, the formalin-fixed and paraffin-embedded samples were deparaffinized with xylene and ethanol for further peroxidase immunohistochemistry using an UltraSensitive^TM^ S-P IHC Kit (MXB Biotechnologies, Cat# Kit-9730). The sections were stained with anti-GSDME (1:100, Proteintech), anti-PMI (1:300, ABclonal) and anti-TUNEL (1:50; Servicebio, Cat# G1507) primary antibodies, and then successively incubated with biotin-labeled anti-mouse/rabbit IgG and peroxidase-conjugated streptavidin. A DAB detection Kit (MXB Biotechnologies, Cat# Kit-0014) was then employed to visualize the stained proteins of interest.

### PAS staining of the kidney

PAS staining was carried out by using a PAS/Glycogen Stain Kit (Nanjing Jiancheng Bioengineering Institute, Cat# D004-1-2). Briefly, the kidneys were fixed and then embedded in paraffin. Kidney sections (5 μm) were deparaffinized, rehydrated, and then stained according to the manufacturer’s protocol.

### AMPK in vitro kinase assay

An AMPK in vitro kinase assay was performed as previously reported.^57^ In brief, Flag-AMPKα1, HA-AMPKβ1 and Myc-AMPKγ1 (4.5 μg:3 μg:3.6 μg in 10-cm dishes) were coexpressed in HEK293T cells, and the AMPK complex was immunoprecipitated with anti-HA antibody. The recombinant proteins in the purified AMPK complex were incubated with bacterially expressed GST-GSDME in 100 μL of kinase buffer (20 mM HEPES, pH 7.4, 5 mM MgCl_2_, 1 mM EGTA, 1 mM DTT, 1% protease and phosphatase inhibitor cocktail, 200 μM ATP, 100 μM AMP and 5 μCi [γ-^32^P]-ATP) at 37 °C for 30 min. The reaction was terminated by the addition of SDS loading buffer. The reaction mixture was then subjected to SDS-PAGE analysis, and autoradiography was performed.

### ITC assay

A MicroCal iTC200 instrument (GE Healthcare) was used to measure the binding affinity between AMPKα1 and substrates at 25 °C. The AMPKα1, AMPKα1^N59A^, AMPKα1^K40A/K42A^, AMPKα1^K40A/K42A/N59A^ and AMPKβ1 proteins were eluted in a buffer containing 25 mM Tris, pH 8.0, 300 mM NaCl, 5 mM MgCl_2_, 1 mM EDTA, 10% glycerol, 2 mM DTT and 10 mM GSH. After that, the proteins were dialyzed with PBS. Substrates were also prepared in PBS. During the titration experiment, the AMPKα1 protein concentration in the cells was 10 μM, while the concentration of GlcNAc-6P as the substrate in the syringe was 1 mM.

### CD spectroscopy

CD measurements were carried out using a Jasco J-810 spectropolarimeter (JASCO, Tokyo). The CD spectra were obtained in 10 mM phosphate buffer (pH 7.4) using a cell with a 0.5-cm path length. 100 μg/mL GSDME^WT^ or GSDME^T6E^ proteins were dialyzed against phosphate buffer (10 mM phosphate, pH 7.4). The 190–260 nm CD spectra were measured. The raw data was analyzed by origin 2019.

### Mannose detection

The mannose concentrations of mouse serum were detected by using the mannose detection Kit (YOUPIN BIOTECH, Cat# YPWB0389), according to the manufacturer’s instructions.

### Mass spectrometry analysis

Phosphorylated GST-GSDME samples were prepared as described in the section describing the in vitro kinase assay with purified AMPK protein. The phosphorylated GST-GSDME protein (4 μg) was subjected to SDS-PAGE and stained with Coomassie blue. The specific band was excised and digested with chymotrypsin before being analyzed by mass spectrometry.

### The in vitro cleavage assay

GSDME-Flag (WT and mutants) was expressed in HEK293T cells and then immunoprecipitated with anti-Flag antibody. Active caspase-3 (1.9 μg/mL) was incubated at 37 °C for 3 h with a GSDME as indicated (WT or mutants) in 10 μL of reaction buffer containing 50 mM HEPES, pH 7.5, 3 mM EDTA, 150 mM NaCl, 0.005% (v/v) Tween-20 and 10 mM DTT. The reaction was terminated by the addition of 10 μL of 2× SDS loading buffer and heating to 95 °C for 10 min.

### Microscopy

Phase-contrast cell images were captured with NIS-Elements software under a Nikon Eclipse Ts2R microscope. For the PI-traced organoid cell death assay, organoids were stained with 5 μg/mL PI for 24 h. For the live-cell imaging of small intestinal organoids, a Cell Discoverer 7 Live Cell Imaging System from Carl Zeiss Microscopy was used. Small intestinal organoid suspensions were cultured in ENR culture medium (with 2 μM Y-27632) and treated with cisplatin (20 μg/mL) and mannose (20 mM) for 12 h.

### LDH release assay

LDH release in cell culture supernatants was analyzed by the CytoTox 96® Non-Radioactive Cytotoxicity Assay Kit (Promega, Cat# G1780) according to the manufacturer’s protocol.

### Cell survival rate

The cell survival rate was measured by staining the cells with PI. In brief, the cells were harvested and resuspended in PBS containing 5 μg/mL PI. The PI-positive cells were quantified by BD Fortessa X20 flow cytometry.

### Cell permeabilization assay

As described previously,^58^ cells were washed once with SLO-permeabilization buffer (137 mM NaCl, 3 mM KCl, 2 mM MgCl_2_, 5.6 mM glucose, 0.1 mg/mL bovine serum albumin, 100 nM CaCl_2_ and 3 mM EGTA, pH 7.2), and fresh SLO-permeabilization buffer containing the indicated metabolite (1 mM) and SLO (200 ng/mL) was added and then incubated at 37 °C for 10 min.

### Metabolite analysis by LC-MS

Cells (5 × 10^6^) were washed three times with PBS (4 °C), and the metabolites in each sample were extracted with 80% methanol solution (1.6 mL, –80 °C). The extract and cells were collected into 2 mL tubes, vortexed for 1 min and centrifuged at 14,000× *g* and 4 °C for 10 min. The supernatants were evaporated to dryness with a vacuum centrifuge (Labconco Corporation). Samples were resuspended in 200 μL of 50% acetonitrile. Each sample (2 μL) was injected and analyzed with a QTRAP (SCIEX, QTRAP 5500) interfaced with a UPLC system (AB Sciex, ExionLC AD system). The sample was loaded onto a ZIC-pHILIC column (SeQuant, 5 μm, 100 × 2.1 mm, Merck). Mobile phase buffer A was 15 mM ammonium acetate (NH_4_Ac, pH adjusted to 9.7 with ammonium hydroxide), and mobile phase buffer B was 90% acetonitrile. During the analysis, the column was maintained at 40 °C, and the sample was maintained at 10 °C. A constant flow rate of 0.2 mL/min was used, and the gradient was as follows: t = 0–2 min, 95% B; t = 15–18 min, 45% B; t = 18–22 min, 95% B. The QTRAP mass spectrometer was run in negative mode with multiple reaction monitoring (MRM) mode. To detect the levels of GlcNAc-6P and GlcNAc-1P, a QTRAP (SCIEX, QTRAP 6500 plus) mass spectrometer interfaced with a UPLC system (Waters, ACQUITY UPLC system) was used. The column was a Luna® NH2 column (5 μm, 50 × 2 mm, Phenomenex). Mobile phase buffer A (pH 9.7) was 20 mM NH_4_Ac, and mobile phase buffer B was 100% acetonitrile. The temperatures of the column and sample were 40 °C and 10 °C, respectively. A constant flow rate of 0.3 mL/min was used, and the gradient was as follows: t = 0–1 min, 90% B; t = 15–17 min, 30% B; t = 17–20 min, 90% B. The QTRAP mass spectrometer was run in negative mode with MRM mode, and the declustering potential and collision energy were optimized using analytical standards. For ^13^C-mannose (Cambridge Isotope Laboratories, Cat# CLM-6567) tracer experiments, A375 cells were treated with 20 mM ^13^C_6_-mannose for 1 h and 6 h.

### Detection of AMPK phosphorylation in the small intestine and kidney

For the small intestine, an appropriate amount of tissue was cut with scissors from anesthetized mice and quickly placed in 2× SDS loading buffer. Then, the tissue was homogenized and boiled. For the kidney, a quarter of the kidney was cut with scissors and placed in 2× SDS loading buffer, followed by tissue homogenization and boiling.

### Small intestinal organoid culture

Small intestinal organoid culture was performed as previously described.^59^ Briefly, C57BL/6 mice at 6–12 weeks of age raised on standard rodent chow and water were sacrificed according to the institutional guidelines. The small intestine was removed, and the contents were flushed with ice-cold 1× PBS (sterile, containing 500 μg/mL gentamicin). The villi were gently scraped away with a sterile glass slide. The crypts were isolated by incubation with 2 mM EDTA at 4 °C for 30 min and passed through a 70-μm cell strainer. The crypts were cultured in Matrigel and advanced DMEM/F12 containing EGF, Noggin and R-spondin1. Noggin and R-spondin1 were produced in HEK293T cells. HEK293T-Noggin and HEK293T-Rspo1 cells were a generous gift from Prof. Dong Gao (Center for Excellence in Molecular Cell Science, Chinese Academy of Sciences). The concentrations of Noggin and R-spondin1 in the conditioned medium were detected by ELISA with anti-Rspo1 antibody and anti-Noggin antibody.

### Statistical analysis

Most of data are expressed as the mean ± SD, and statistical analysis of differences between two groups was performed using two-tailed Student’s *t*-test. Differences between multiple groups were analyzed using one-way or two-way ANOVA, followed by Tukey’s or Sidak’s multiple-comparisons test. For clinical data, numbers of BMs and other indexes from feces and blood were expressed as the mean and calculated with 95% confidence intervals (CIs), and statistical analysis of numbers of BMs was performed by using two-way ANOVA while indexes from feces and blood were calculated with two-tailed Student’s *t*-test. Statistical analysis was performed using GraphPad Prism 7. *P < 0.05 indicated a statistically significant difference, **P < 0.01 indicated a highly significant difference, and ***P < 0.001 indicated an extremely significant difference.

### Supplementary information

Supplementary informention, Fig. S1

Supplementary informention, Fig. S2

Supplementary informention, Fig. S3

Supplementary informention, Fig. S4

Supplementary informention, Fig. S5

Supplementary informention, Fig. S6

Supplementary informention, Fig. S7

Supplementary informention, Fig. S8

Supplementary information, Table S1

Supplementary information, Table S2

Supplementary information, Video S1

Supplementary information, Video S2

Supplementary Video legends
