# Supplementary material for: Mannose antagonizes GSDME-mediated pyroptosis through AMPK activated by metabolite GlcNAc-6P
Source: Cell Res. 2023 Jul 17;33(12):904–22. doi: 10.1038/s41422-023-00848-6 (PMC10709431; doi:10.1038/s41422-023-00848-6)
Supplement: Supplementary file 1 — Supplementary informention, Fig. S1 [file 41422_2023_848_MOESM1_ESM.pdf]

Supplementary information, Fig. S1

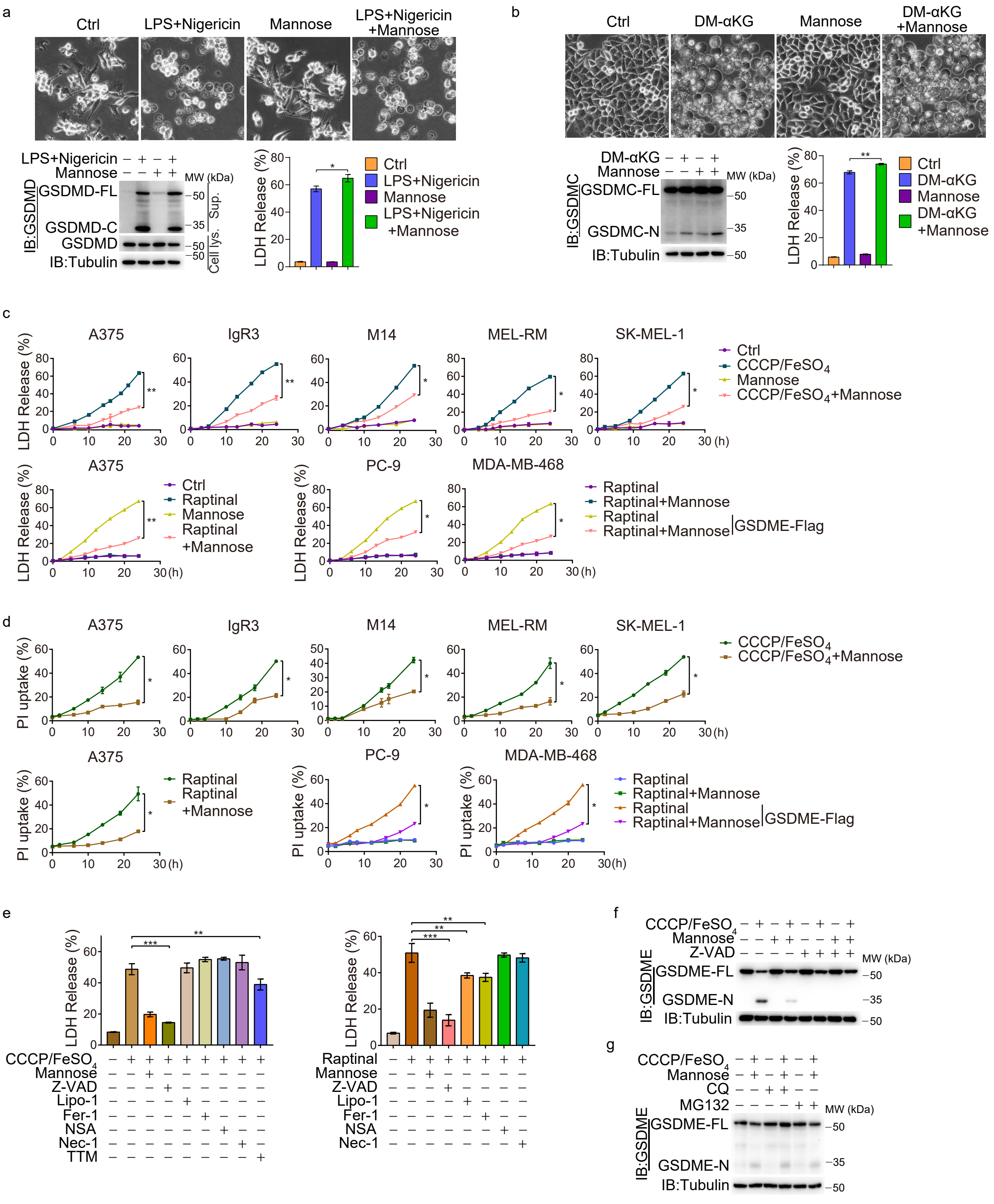

Supplementary information, Fig. S1. **a** Mannose has no effect on LPS and nigericin-induced GSDMD-mediated pyroptosis. THP-1 cells were primed with PMA (50 nM) for 36 hours, and then pretreated with mannose for 2 hours, followed by LPS (100 ng/ml) for 4 hours and finally by nigericin (10 μM) for 2 hours. Pyroptosis was detected. **b** Mannose has no effect on DM-αKG-induced GSDMC-mediated pyroptosis. HeLa cells were pretreated with mannose for 2 hours, and then treated with DM-αKG (15 mM) for 24 hours. Pyroptosis was detected. **c,d** The kinetics of LDH release and PI uptake were shown for the different treatments as indicated in different cancer cell lines. **e** A375 cells were pretreated with mannose (20 mM), Z-VAD (20 μM), Lipo-1 (0.5 μM), Fer-1 (0.5 μM), NSA (5 μM), Nec-1 (20 μM), or TTM (20 μM) as indicated for 2 hours, and then treated with CCCP/$FeSO_4$ or raptinal for 24 hours. LDH release were detected. **f** Z-VAD showed no effect on the stability of GSDME-FL in the presence of CCCP/$FeSO_4$ with or without mannose cotreatment. **g** A375 cells were pretreated with CQ (30 μM) or MG132 (100 nM) for 2 hours, and then treated with CCCP/$FeSO_4$ plus mannose for 24 hours. Tubulin was used to determine the amount of loading proteins. All data are presented as the mean ± SD of two independent experiments, and one of western blotting results is presented. ***P<0.001, **P<0.01, *P<0.05.
