# Supplementary material for: Mannose antagonizes GSDME-mediated pyroptosis through AMPK activated by metabolite GlcNAc-6P
Source: Cell Res. 2023 Jul 17;33(12):904–22. doi: 10.1038/s41422-023-00848-6 (PMC10709431; doi:10.1038/s41422-023-00848-6)
Supplement: Supplementary file 2 — Supplementary informention, Fig. S2 [file 41422_2023_848_MOESM2_ESM.pdf]

Supplementary information, Fig. S2

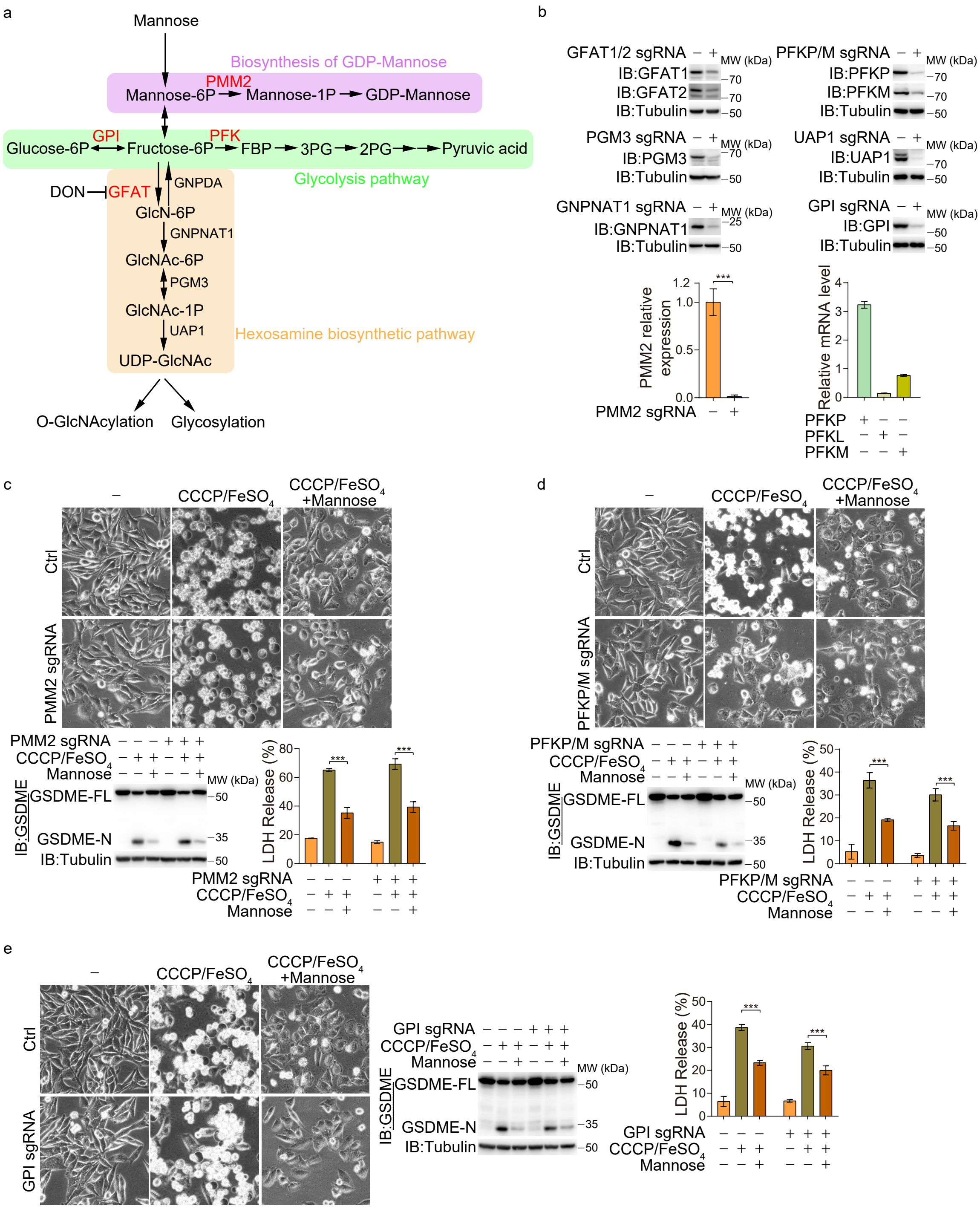

Supplementary information, Fig. S2. In this figure, melanoma A375 cells were pretreated with mannose (20 mM) for 2 hours, and then CCCP/$FeSO_4$ (CCCP 20 μM, $FeSO_4$ 100 μM) for 24 hours to assess pyroptosis (including characteristic morphology, GSDME cleavage, and LDH release), unless specially defined. **a** Schematic diagram of mannose metabolism pathways. **b** Efficiencies of knocking out different genes and mRNA expression of PFK isoforms in cells. **c-e** Knocking out PMM2 (**c**) and GPI (**e**) or double knocking out PFKP/M (**d**) did not affect the suppressive function of mannose in CCCP/$FeSO_4$-induced pyroptosis. These genes were separately knocked out in cells first, and the cells were treated with CCCP/$FeSO_4$ with or without mannose cotreatment, pyroptosis was then determined. Tubulin was used to determine the amount of loading proteins. All data are presented as the mean ± SD of two independent experiments, and one of western blotting results is presented. ***P<0.001.
