# Supplementary material for: Mannose antagonizes GSDME-mediated pyroptosis through AMPK activated by metabolite GlcNAc-6P
Source: Cell Res. 2023 Jul 17;33(12):904–22. doi: 10.1038/s41422-023-00848-6 (PMC10709431; doi:10.1038/s41422-023-00848-6)
Supplement: Supplementary file 3 — Supplementary informention, Fig. S3 [file 41422_2023_848_MOESM3_ESM.pdf]

Supplementary information, Fig. S3

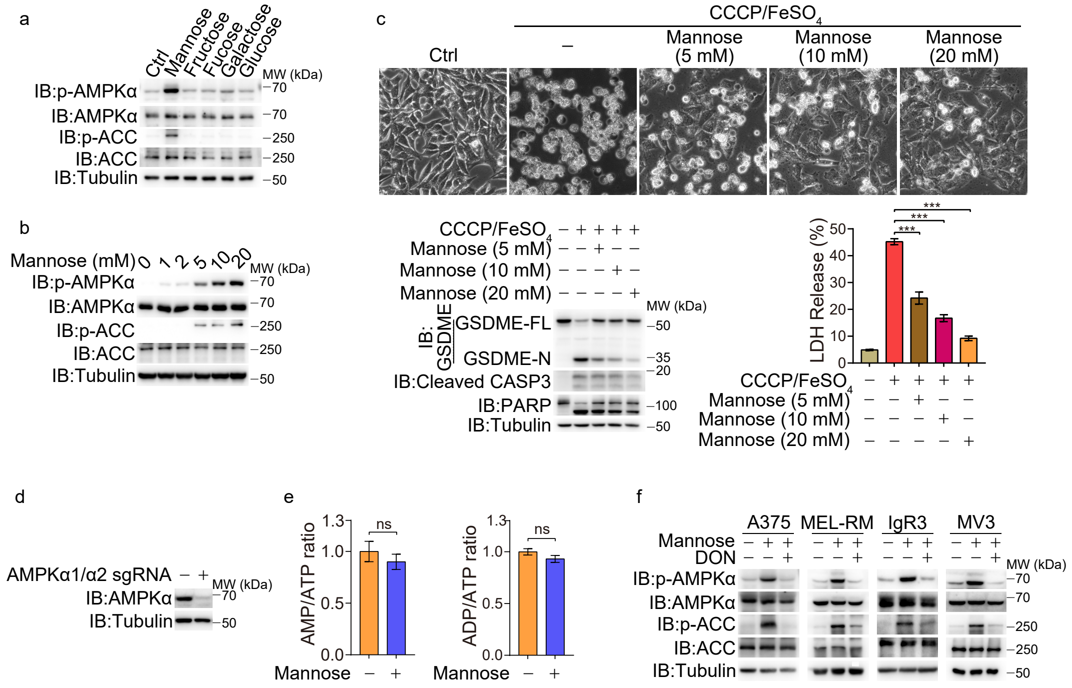

Supplementary information, Fig. S3. a Mannose, but not other hexoses, induced AMPK and ACC phosphorylation. A375 cells were treated with different hexoses as indicated (concentrations used are the same as Fig. 1d) for 6 hours. The phosphorylation levels of AMPK (Thr172) and ACC (Ser79) were detected. **b** A375 cells were treated with mannose at indicated doses for 6 hours, and the phosphorylation levels of AMPK and ACC were detected. **c** A375 cells were treated with CCCP/$FeSO_4$ with or without mannose at indicated doses for 24 hours, pyroptosis was detected. **d** Efficiency of double knocking out AMPKα1/α2 genes in A375 cells. **e** Mannose has no effect on AMP/ATP or ADP/ATP ratio. A375 cells were treated with mannose for 6 hours, the ratio of AMP/ATP or ADP/ATP was detected. **f** DON attenuated mannose-activated AMPK phosphorylation in melanoma cell lines. Different cell lines as indicated were pretreated with DON (40 μM) for 2 hours, and then mannose for 6 hours. The AMPK and ACC phosphorylation were detected. Tubulin was used to determine the amount of loading proteins. All data are presented as the mean ± SD of two independent experiments, and one of western blotting results is presented. ***$P<0.001$; ns, not significant.
