# Supplementary material for: Mannose antagonizes GSDME-mediated pyroptosis through AMPK activated by metabolite GlcNAc-6P
Source: Cell Res. 2023 Jul 17;33(12):904–22. doi: 10.1038/s41422-023-00848-6 (PMC10709431; doi:10.1038/s41422-023-00848-6)
Supplement: Supplementary file 4 — Supplementary informention, Fig. S4 [file 41422_2023_848_MOESM4_ESM.pdf]

Supplementary information, Fig. S4

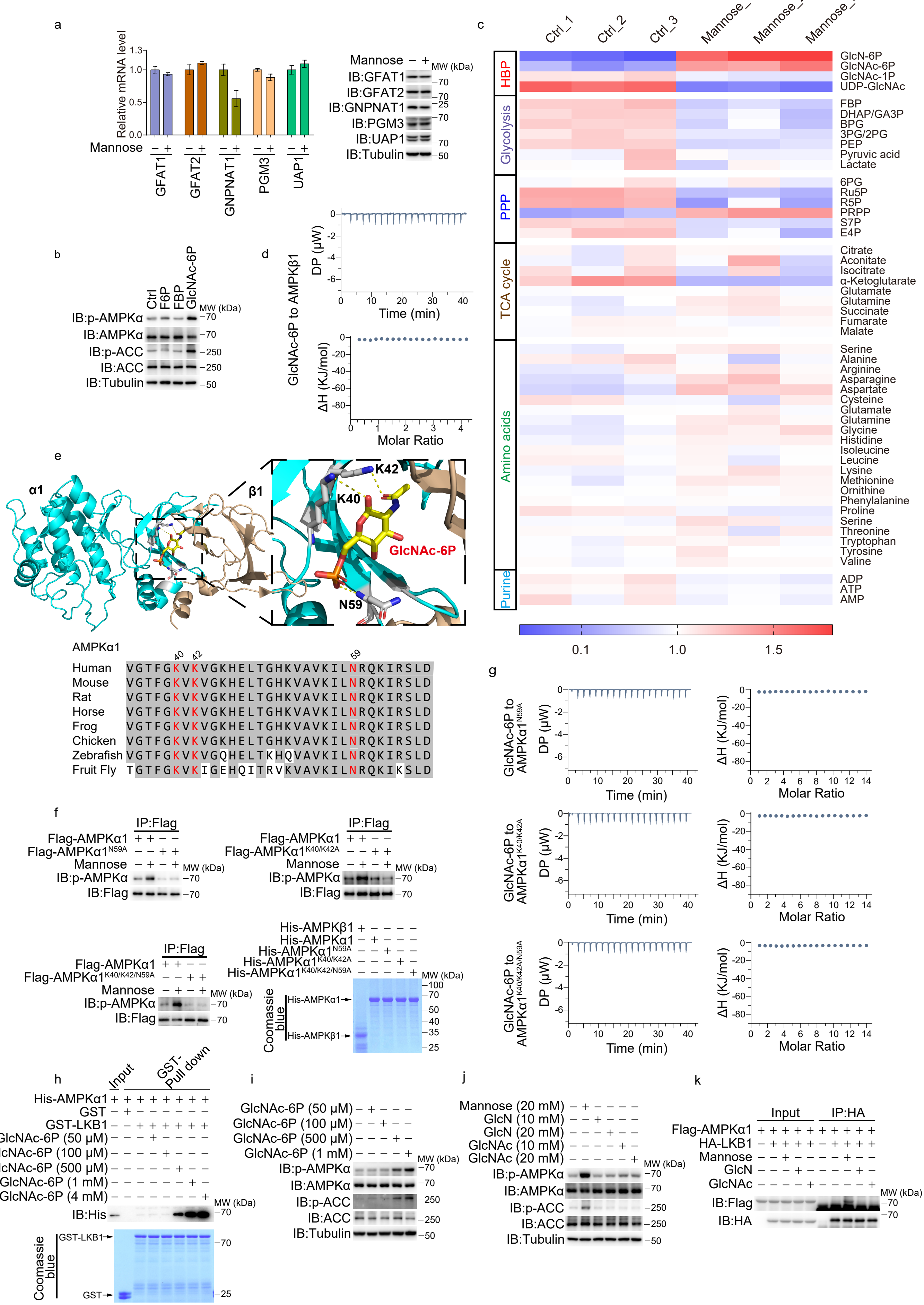

l

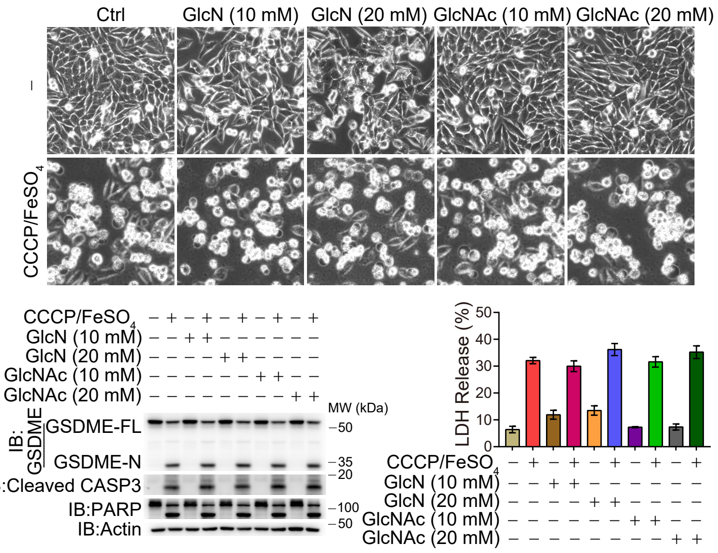
Ctrl
GlcN (10 mM)
GlcN (20 mM)
GlcNAc (10 mM)
GlcNAc (20 mM)
–
CCCP/FeSO4
CCCP/FeSO4
GlcN (10 mM)
GlcN (20 mM)
GlcNAc (10 mM)
GlcNAc (20 mM)
MW (kDa)
IB: GSDME
GSDME-FL
GSDME-N
IB:Cleaved CASP3
IB:PARP
IB:Actin
LDH Release (%)
50
40
30
20
10
0

m

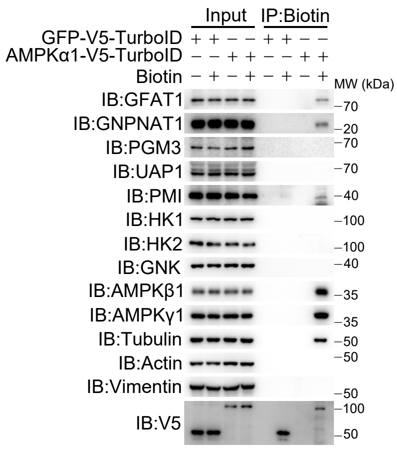
Input
IP:Biotin
GFP-V5-TurboID
AMPKα1-V5-TurboID
Biotin
MW (kDa)
IB:GFAT1
IB:GNPNAT1
IB:PGM3
IB:UAP1
IB:PMI
IB:HK1
IB:HK2
IB:GNK
IB:AMPKβ1
IB:AMPKγ1
IB:Tubulin
IB:Actin
IB:Vimentin
IB:V5

n

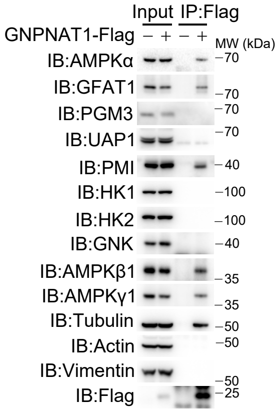
Input
IP:Flag
GNPNAT1-Flag
MW (kDa)
IB:AMPKα
IB:GFAT1
IB:PGM3
IB:UAP1
IB:PMI
IB:HK1
IB:HK2
IB:GNK
IB:AMPKβ1
IB:AMPKγ1
IB:Tubulin
IB:Actin
IB:Vimentin
IB:Flag

o

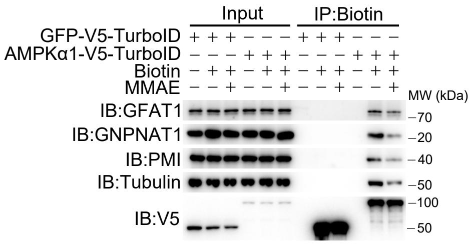
Input
IP:Biotin
GFP-V5-TurboID
AMPKα1-V5-TurboID
Biotin
MMAE
MW (kDa)
IB:GFAT1
IB:GNPNAT1
IB:PMI
IB:Tubulin
IB:V5

p

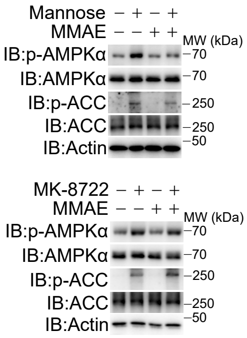
Mannose
MMAE
MW (kDa)
IB:p-AMPKα
IB:AMPKα
IB:p-ACC
IB:ACC
IB:Actin
MK-8722
MMAE
MW (kDa)
IB:p-AMPKα
IB:AMPKα
IB:p-ACC
IB:ACC
IB:Actin

Supplementary information, Fig. S4. a Mannose had no effects on the mRNA and protein expression levels of GFAT1/2, GNPNAT1, PGM3 and UAP1. A375 cells were treated with mannose (20 mM) for 6 hours, and then the mRNA and protein expression levels were detected by RT-PCR and western blotting. **b** F6P and FBP could not induce AMPK phosphorylation. SLO (200 ng/ml) and different metabolites (1 mM) as indicated were incubated with A375 cells for 10 minutes. The phosphorylation levels of AMPK and ACC were detected. **c** Heatmap depicts the metabolite intensities in the metabolomics data. A375 cells were treated with mannose (20 mM) for 6 hours. **d** Metabolite GlcNAc-6P does not bind to AMPKβ1, detected by ITC assay. **e** Docking analysis indicates the possible sites of GlcNAc-6P binding to AMPK (top). Sequence alignment of AMPKα1 from several species. The alignment was abtained from T-COFFEE algorithm. All species contain K40, K42 and N59 residues (highlighted red)(bottom). **f** Different point mutations as indicated attenuated mannose-induced AMPK phosphorylation. A375 cells were transfected with AMPKα1 with different point mutations, and then treated with mannose (20 mM) for 6 hours, AMPK phosphorylation was detected. **g** GlcNAc-6P does not bind to three mutants of AMPKα1 detected by ITC assay. **h,i** Effects of different doses of GlcNAc-6P on the interaction of AMPKα1-LKB1 (**h**) and AMPK phosphorylation (**i**). His-AMPKα1 and GST-LKB1 extracted from bacteria were incubated with different doses of GlcNAc-6P as indicated (**h**). A375 cells were incubated with different doses of GlcNAc-6P, the AMPK and ACC phosphorylation were detected (**i**). **j** Effects of different doses of GlcN and GlcNAc on AMPK phosphorylation. Mannose (20 mM) or different metabolites (10 mM or 20 mM) were incubated with A375 cells for 6 hours. The phosphorylation levels of AMPK and ACC were detected. **k** GlcN and GlcNAc showed no effects on AMPKα1-LKB1 interaction. A375 cells were transfected with AMPKα1 and then treated with mannose (20 mM), GlcN (20 mM) or GlcNAc (20 mM) for 6 hours, the AMPKα1-LKB1 interaction was detected. **l** GlcN and GlcNAc showed no effects on CCCP/$FeSO_4$-induced pyroptosis. A375 cells were pretreated with different doses of GlcN or GlcNAc for 2 hours, and then CCCP/$FeSO_4$ (CCCP 20 μM, $FeSO_4$ 100 μM) for 24 hours to assess pyroptosis. **m,n** TurboID (**m**) and co-IP (**n**) assays showed the complex of GFAT1, GNPNAT1, Tubulin and AMPK. A375 cells were transfected with AMPKα1-turboID and then treated with biotin (100 μM) for 10 min, biotin-labeled proteins were isolated (**m**).

Different plasmids were transfected into A375 cells and then co-IP assay was performed (**n**). **o** MMAE impaired complex formation of GFAT1, GNPNAT1, tubulin and AMPK. A375 cells were pretreated with MMAE (1 μg/mL) for 1 hour before treated with biotin. **p** Inhibitory effect of MMAE on mannose-induced AMPK phosphorylation. A375 cells were pretreated with MMAE (1 μg/mL) for 1 hour, and then mannose or MK-8722 for 6 hours. AMPK and ACC phosphorylation were detected. Tubulin or actin was used to determine the amount of loading proteins. All data are presented as the mean ± SD of two independent experiments, and one of western blotting results is presented.
