# Supplementary material for: Mannose antagonizes GSDME-mediated pyroptosis through AMPK activated by metabolite GlcNAc-6P
Source: Cell Res. 2023 Jul 17;33(12):904–22. doi: 10.1038/s41422-023-00848-6 (PMC10709431; doi:10.1038/s41422-023-00848-6)
Supplement: Supplementary file 5 — Supplementary informention, Fig. S5 [file 41422_2023_848_MOESM5_ESM.pdf]

Supplementary information, Fig. S5

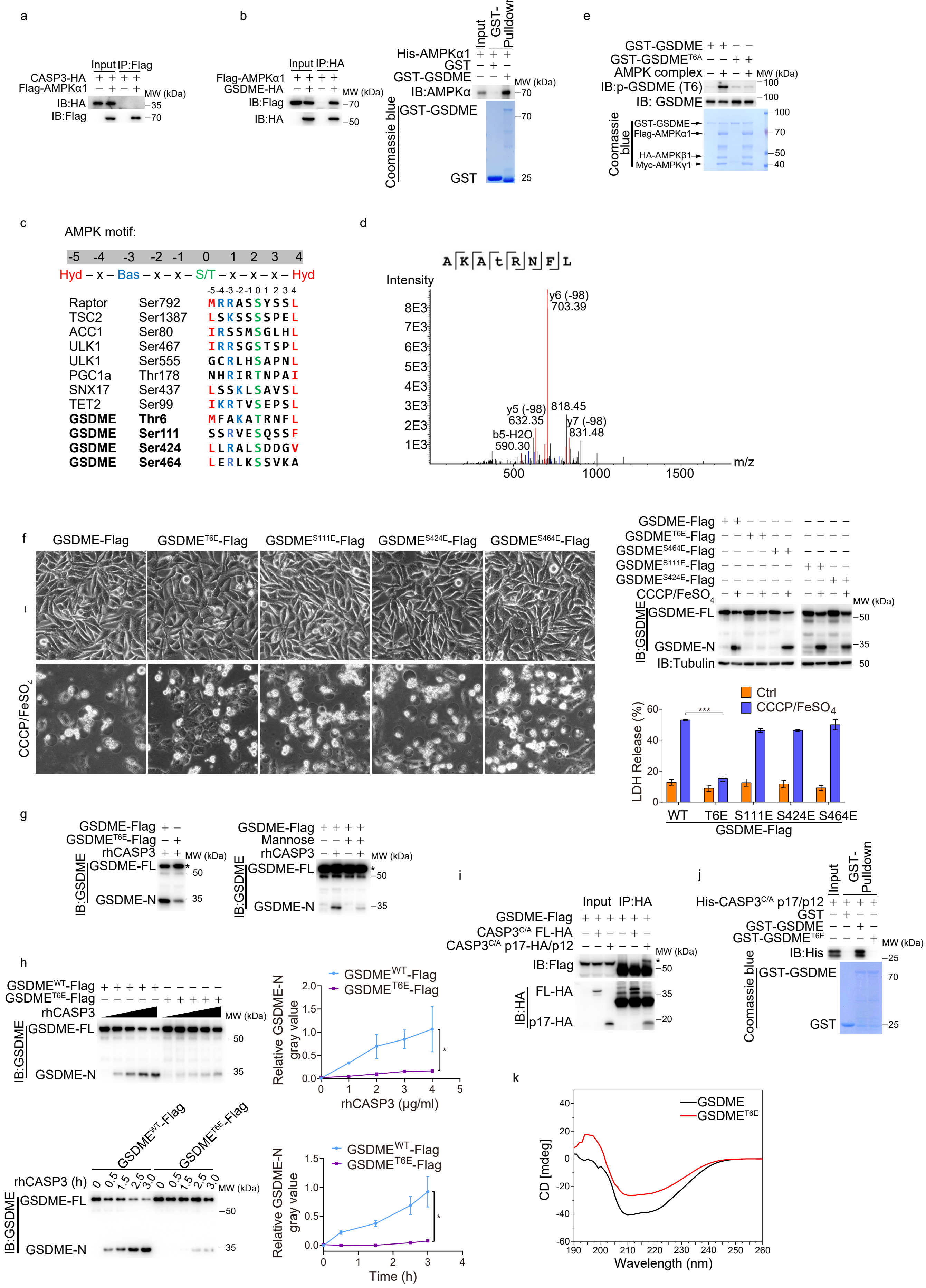

Supplementary information, Fig. S5. a Caspase-3 could not interact with AMPKα1. Caspase-3 and AMPKα1 were transfected into A375 cells, co-IP assay was performed. **b** GSDME interacted with AMPKα1, detected by co-IP assay (left) and GST pulldown assay (right). Left, GSDME and AMPKα1 were transfected into A375 cells. Right, His-AMPKα1 and GST-GSDME extracted from bacteria were incubated. **c** Reported AMPK motif of several AMPK target proteins and putative motif of GSDME. **d** Mapping the AMPK phosphorylation of Thr6 in GSDME in the *in vitro* assay by mass spectrometry. **e** AMPK directly phosphorylated GSDME detected in the *in vitro* AMPK kinase assay. Reactions were resolved by SDS-PAGE and detected by Phospho-GSDME (T6) antibody. **f** Different GSDME point mutants affect CCCP/$FeSO_4$-induced pyroptosis. Different GSDME point mutants as indicated were transfected into GSDME knockout A375 cells, the cells were then treated with CCCP/$FeSO_4$ (CCCP 20 μM, $FeSO_4$ 100 μM) for 24 hours, pyroptotic features were detected. **g** Determination of GSDME cleavage. GSDME or $GSDME^{T6E}$ was separately transfected into HEK293T cells (left), or GSDME was transfected into A375 cells that were then treated with mannose (20 mM) for 6 hours (right), expressed proteins were isolated and then incubated with recombinant human caspase-3 protein. The cleavage of GSDME was showed. The asterisk indicates GSDME-Flag. **h** GSDME or $GSDME^{T6E}$ was separately transfected into HEK293T cells, and then expressed proteins were isolated to incubate with recombinant human caspase-3 protein at different doses and times. The cleavage of GSDME and the relative GSDME-N gray value were shown. **i** Determination of GSDME interaction with caspase-3 catalytically inactive mutant ($CASP3^{C/A}$p17-HA/p12) but not with full-length caspase-3 ($CASP3^{C/A}$FL-HA). The asterisk indicates co-IP GSDME-Flag. **j** Interaction of caspase-3 catalytically inactive mutant (His-$CASP3^{C/A}$p17/p12) with GSDME or $GSDME^{T6E}$ *in vitro*. GST pulldown was performed. **k** Conformation patterns between GSDME and $GSDME^{T6E}$. GST-GSDME and GST-$GSDME^{T6E}$ extracted from bacteria were analyzed by Jasco J-810 spectropolarimeter (JASCO, Tokyo). Tubulin was used to determine the amount of loading proteins. All data are presented as the mean ± SD of two independent experiments, and one of western blotting results is presented. ***P<0.001, *P<0.05.
