# Supplementary material for: Mannose antagonizes GSDME-mediated pyroptosis through AMPK activated by metabolite GlcNAc-6P
Source: Cell Res. 2023 Jul 17;33(12):904–22. doi: 10.1038/s41422-023-00848-6 (PMC10709431; doi:10.1038/s41422-023-00848-6)
Supplement: Supplementary file 6 — Supplementary informention, Fig. S6 [file 41422_2023_848_MOESM6_ESM.pdf]

Supplementary information, Fig. S6

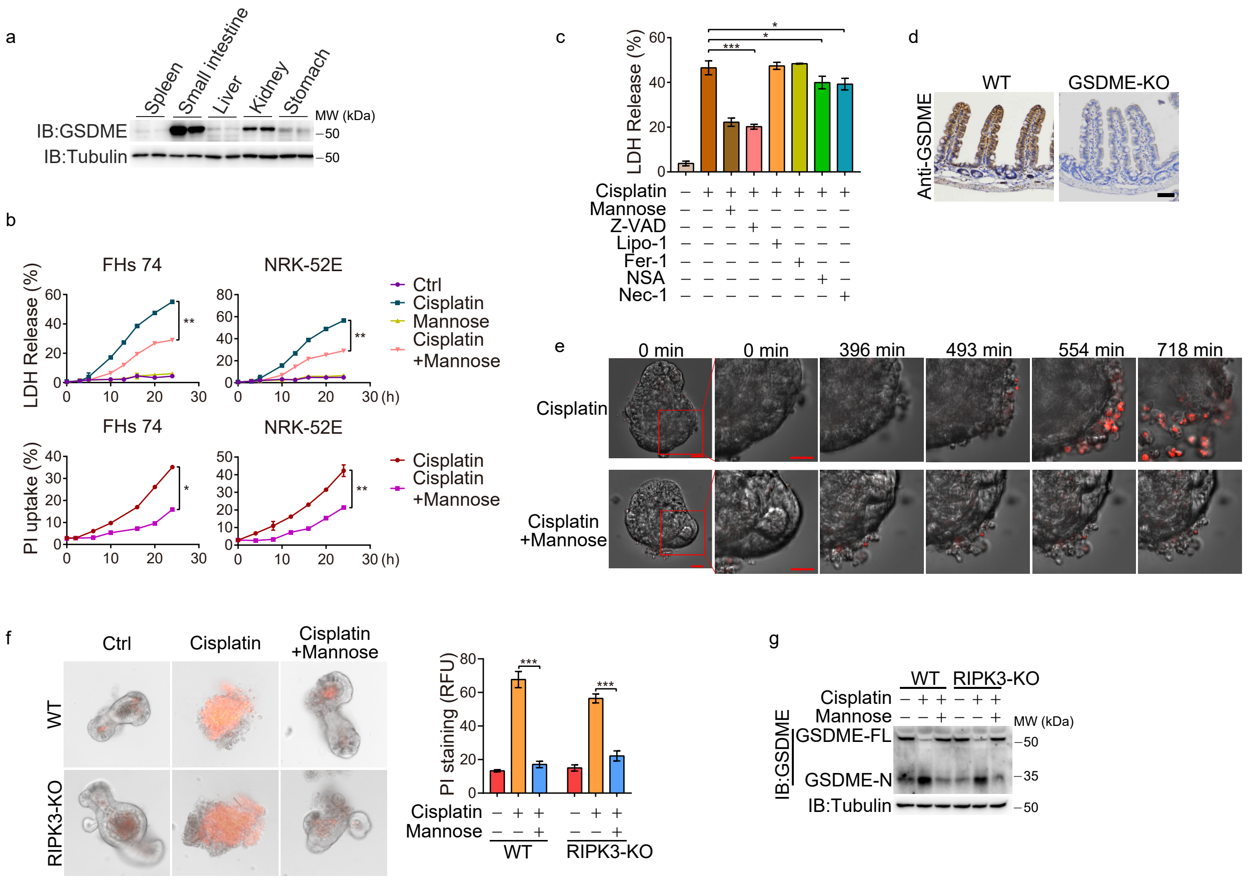

Supplementary information, Fig. S6. a Determination of the expression levels of GSDME in different organs. Different organs from mice were collected and lysed. The expression levels of GSDME in different organs were determined by western blotting. **b** The kinetics of LDH release and PI uptake were shown with the different treatments as indicated in FHs 74 and NRK-52E cells. **c** FHs 74 cells were pretreated with mannose (20 mM), Z-VAD (20 μM), Lipo-1 (0.5 μM), Fer-1 (0.5 μM), NSA (5 μM), or Nec-1 (20 μM) as indicated for 2 hours, and then treated with cisplatin for 24 hours. LDH release was detected. **d** Efficiency of knocking-out GSDME in small intestine. **e**, Time-lapse live cell image of small intestinal organoid. Pyroptotic cell death was indicated by PI staining. Several images were taken at the indicated time points after cisplatin treatment. Real-time videos are included in Supplementary information, Videos S1-2. **f** Mannose reversed cisplatin-induced pyroptotic cell death indicated by PI staining in wild type and RIPK3 knockout small intestinal organoid. **g** Mannose inhibited cisplatin-induced GSDME cleavage in wild type and RIPK3 knockout small intestinal organoid. ***P<0.001, **P<0.01, *P<0.05.
