# Supplementary material for: Mannose antagonizes GSDME-mediated pyroptosis through AMPK activated by metabolite GlcNAc-6P
Source: Cell Res. 2023 Jul 17;33(12):904–22. doi: 10.1038/s41422-023-00848-6 (PMC10709431; doi:10.1038/s41422-023-00848-6)
Supplement: Supplementary file 7 — Supplementary informention, Fig. S7 [file 41422_2023_848_MOESM7_ESM.pdf]

Supplementary information, Fig. S7

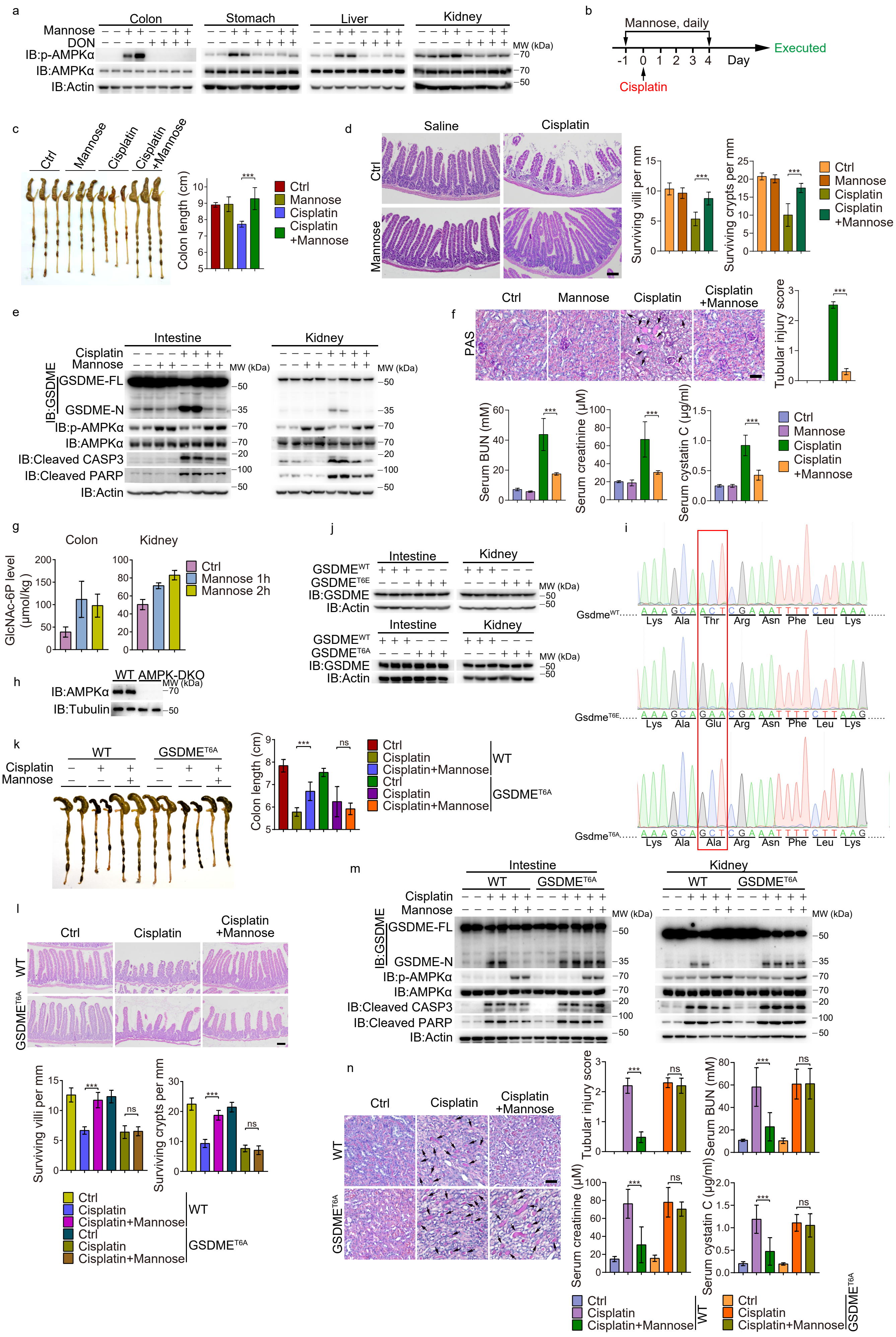

Supplementary information, Fig. S7. a Mice were administrated with mannose for four days with or without DON (20 mg/Kg) treatment, AMPK phosphorylation in different tissues were detected. **b** Schematic diagram of mannose together with cisplatin administration in normal mice. **c,d** Mannose rescued cisplatin-induced damages of intestine, including colon shorten (**c**), loss of crypts, and the reduced villus lengths (**d**; scale bar, 100 μm) (n=5). **e** Mannose abolished cisplatin-induced GSDME cleavage, cleaved caspase-3 and its substrate PARP, while elevated AMPK phosphorylation in small intestine (left) and kidney (right). **f** Mannose rescued cisplatin-induced damages of kidney, indicated by periodic acid-Schiff (PAS) staining (top, the injured tubules were indicated by arrows (left) and quantified (right). Scale bar, 100 μm), and the levels of serum BUN, serum creatinine, and serum cystatin C (bottom). **g** The level of GlcNAc-6P in colon and kidney detected by LC-MS. **h** Efficiency of knocking out AMPK in small intestinal organoid. The small intestinal organoids were constructed from AMPK-DKO mouse model. **i** Genotype of $GSDME^{T6E}$ and $GSDME^{T6A}$ mice was identified by sequencing. **j** Determination of GSDME expression levels in intestine and kidney of $GSDME^{T6E}$ and $GSDME^{T6A}$ transgenic mice by western blotting. **k-n** In $GSDME^{T6A}$ knock-in mice (n=6) , cisplatin exerted its effects on inducing damages of small intestine and kidney, including colon shorten (**k**), loss of crypts and the reduced villus lengths (**l**), damages of kidney (**n**, left), increasing the levels of serum BUN, serum creatinine, and serum cystatin C (**n**, right), and cleavage of GSDME, cleaved caspase-3 and its substrate PARP in both intestine and kidney (**m**). Tubulin or actin was used to determine the amount of loading proteins. All data are presented as the mean ± SD of two independent experiments, and one of western blotting results is presented. ***P<0.001; ns, not significant.
