# Supplementary material for: Mannose antagonizes GSDME-mediated pyroptosis through AMPK activated by metabolite GlcNAc-6P
Source: Cell Res. 2023 Jul 17;33(12):904–22. doi: 10.1038/s41422-023-00848-6 (PMC10709431; doi:10.1038/s41422-023-00848-6)
Supplement: Supplementary file 8 — Supplementary informention, Fig. S8 [file 41422_2023_848_MOESM8_ESM.pdf]

Supplementary information, Fig. S8

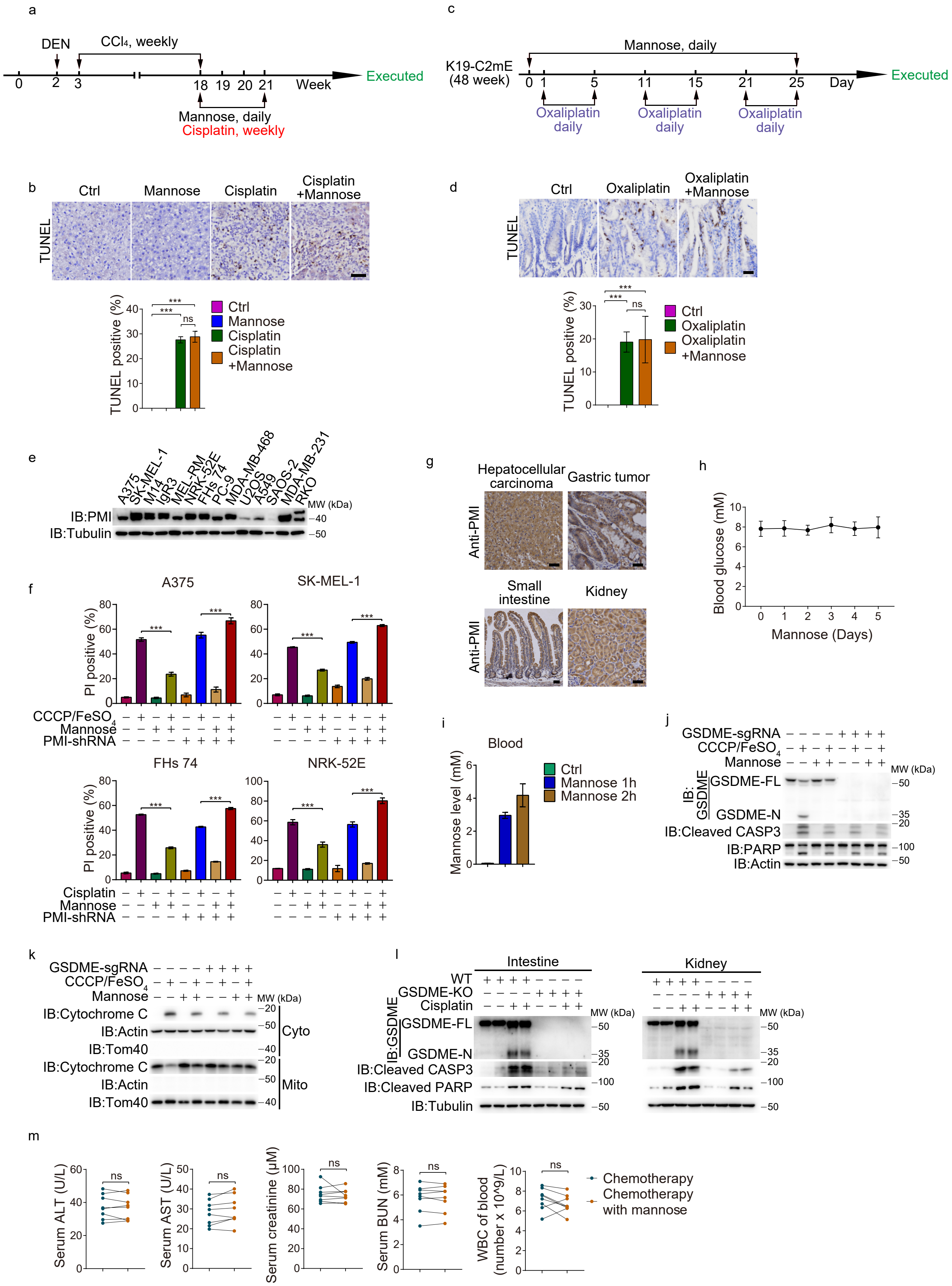

Supplementary information, Fig. S8. a Schematic diagram of mannose together with cisplatin administration in DEN/CCl_4-induced liver cancer model. **b** TUNEL staining indicated cisplatin-induced apoptosis and mannose showed no effect on apoptosis induction, detected in liver cancer samples. **c** Schematic diagram of mannose together with oxaliplatin administration in the gastric mucosa specific expression of COX-2/mPGES-1 transgenic mouse model. **d** TUNEL staining indicated oxaliplatin-induced apoptosis and mannose showed no effect on apoptosis induction, detected in gastric carcinoma samples. **e** Determination of the expression levels of PMI in different cell lines. Cells were collected and lysed. The expression levels of PMI were determined by western blotting. **f** In PMI knockdown A375 cells, SK-MEL-1 cells, FHs 74 cells and NRK-52E cells, mannose (20 mM) was used to pretreated cells for 2 hours, followed by CCCP/$FeSO_4$ (A375 and SK-MEL-1) or cisplatin (FHs 74 and NRK-52E) respectively, LDH release was then detected. **g** Immunohistochemical (IHC) analysis of PMI in hepatocellular carcinoma tissue in DEN/CCl_4-induced liver tumor model, and in gastric tumor tissue in K19-C2mE gastric tumor model, as well as in small intestine and kidney tissues in normal mice. **h** Mannose could not elevate the levels of blood glucose at indicated times of mice (n=10). **i** Detection of mannose in blood. **j,k** In GSDME knockout A375cells, mannose (20 mM) was used to pretreated cells for 2 hours, followed by CCCP/$FeSO_4$, cleaved caspase-3 and its substrate PARP levels were shown (**j**), the release of cytochrome C from mitochondria to cytosol was also shown (**k**). **l** In WT and GSDME-KO mice, the cleaved caspase-3 and its substrate PARP levels in small intestine (left) and kidney (right) were detected after treated with cisplatin. **m** The biochemical examination includes the levels of alanine transaminase (ALT), Aspartate transferase (AST), serum creatinine (CR), blood urea nitrogen (BUN) and WBC detected in the serum. All data are presented as the mean ± SD of two independent experiments. ***P<0.001; ns, not significant.
