# Supplementary material for: Mannose antagonizes GSDME-mediated pyroptosis through AMPK activated by metabolite GlcNAc-6P
Source: Cell Res. 2023 Jul 17;33(12):904–22. doi: 10.1038/s41422-023-00848-6 (PMC10709431; doi:10.1038/s41422-023-00848-6)
Supplement: Supplementary file 13 — Supplementary Video legends [file 41422_2023_848_MOESM13_ESM.pdf]

Supplementary information, Video S1 Time-lapse analysis for pyroptosis of small intestinal organoids treated with cisplatin. Small intestinal organoids were treated with cisplatin (20 μg/mL) for 12 h. Propidium iodide (5 μg/mL, red) was used to indicate the occurrence of pyroptosis.

Supplementary information, Video S2 Time-lapse analysis for pyroptosis of small intestinal organoids treated with cisplatin and mannose. Small intestinal organoids were treated with cisplatin (20 μg/mL) and mannose (20 mM) for 12 h. Propidium iodide (5 μg/mL, red) was used to indicate the occurrence of pyroptosis.
